# Combinatorial histone modifications direct ATP-dependent chromatin remodeling by NURF to promoter-proximal nucleosomes

**DOI:** 10.1093/nar/gkag494

**Published:** 2026-05-21

**Authors:** So Yeon Kwon, Boyun Jang, Valentina Grisan, Yutong Liang, Michelle A C Reed, Hyeim Jung, Ye Eun Lee, Robyn Halston, Ka Hei Chan, Giovanni Bottegoni, Ulrich L Gunther, Carl Wu, Paul Badenhorst

**Affiliations:** Department of Cancer and Genomic Sciences, School of Medical Sciences, College of Medicine and Health, University of Birmingham, Edgbaston B15 2TT, United Kingdom; Department of Cancer and Genomic Sciences, School of Medical Sciences, College of Medicine and Health, University of Birmingham, Edgbaston B15 2TT, United Kingdom; Department of Cancer and Genomic Sciences, School of Medical Sciences, College of Medicine and Health, University of Birmingham, Edgbaston B15 2TT, United Kingdom; Department of Cancer and Genomic Sciences, School of Medical Sciences, College of Medicine and Health, University of Birmingham, Edgbaston B15 2TT, United Kingdom; Department of Cancer and Genomic Sciences, School of Medical Sciences, College of Medicine and Health, University of Birmingham, Edgbaston B15 2TT, United Kingdom; Department of Cancer and Genomic Sciences, School of Medical Sciences, College of Medicine and Health, University of Birmingham, Edgbaston B15 2TT, United Kingdom; Department of Cancer and Genomic Sciences, School of Medical Sciences, College of Medicine and Health, University of Birmingham, Edgbaston B15 2TT, United Kingdom; Department of Cancer and Genomic Sciences, School of Medical Sciences, College of Medicine and Health, University of Birmingham, Edgbaston B15 2TT, United Kingdom; Department of Cancer and Genomic Sciences, School of Medical Sciences, College of Medicine and Health, University of Birmingham, Edgbaston B15 2TT, United Kingdom; Department of Biomolecular Sciences, Università degli Studi di Urbino Carlo Bo, Via Aurelio Saffi 2, 61029 Urbino PU, Italy; Henry Wellcome Building for Biomolecular NMR Spectroscopy, School of Cancer Sciences, University of Birmingham, Edgbaston B15 2TT, United Kingdom; Institut für Chemie und Metabolomics, Universität zu Lübeck, 23562 Lübeck, Germany; Department of Biology, Johns Hopkins University, 3400 N. Charles Street, Baltimore, MD 21218, United States; Department of Cancer and Genomic Sciences, School of Medical Sciences, College of Medicine and Health, University of Birmingham, Edgbaston B15 2TT, United Kingdom

## Abstract

The nucleosome remodeling factor (NURF) is a conserved imitation switch-containing ATP-dependent chromatin remodeling complex that slides nucleosomes to control transcription and genome organization. Recognition of histone modifications (HPTMs) by reader domains has been proposed to focus remodeler action at discrete genome targets, either by controlling recruitment or through local allosteric regulation of core enzymatic activities. To distinguish mechanisms by which HPTMs influence NURF, we defined the HPTM-binding specificity of NURF by screening novel combinations of histone-reader interactions. We observe the NURF-selective subunit (BPTF/NURF301) recognizes multiple H3 and H4 tail modifications via C-terminal PHD2 and bromodomains. Modified H3 recognition requires a new binding-pocket on PHD2 for H3K9AcS10p that cooperates with the known H3K4me3-binding hydrophobic cage to enable high-affinity binding to triply modified H3K4me3K9AcS10p. This combinatorial HPTM recognition discriminates and stabilizes NURF recruitment to +1 nucleosomes of active genes, maintaining nucleosome position to control transcription. Our data establish direct, causal links between HPTM recognition, remodeler recruitment, and consequent activity.

## Graphical Abstract

**Graphical Abstract:**
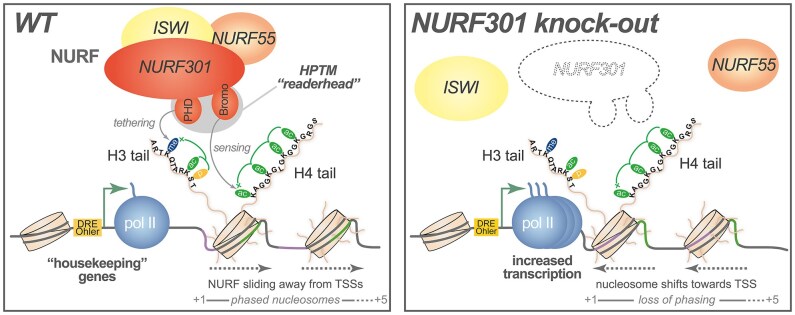

## Introduction

Nucleosome position and dynamics profoundly modulate DNA transactions, including transcription, replication, and repair, by controlling access to DNA of the molecular complexes that process genetic information. By varying nucleosome organization, targets for the transcription, replication, and repair machineries can be rendered inaccessible or made available for activity. Post-translational modification of the histone tails (HPTMs) provides one route by which associations between histones and DNA, and between neighbouring nucleosomes, can be altered to regulate chromatin flexibility and conformation (reviewed in [1, 2]). However, these modifications can also be bound or influence the activity of effector complexes that include energy-dependent chromatin remodeling factors.

ATP-dependent chromatin remodeling factors are large multi-subunit molecular machines that utilize the energy of ATP hydrolysis to slide nucleosomes, alter histone variant composition, or potentially evict nucleosomes (reviewed in [3–5]). The imitation switch (ISWI) chromatin remodelers mediate energy-dependent nucleosome sliding [6, 7] and include the conserved nucleosome remodeling factor (NURF). Our previous investigations have indicated broad roles for NURF in transcription of targets ranging from homeotic selector genes to steroid-responsive targets, and a subset of JAK/STAT inflammatory promoters [8–10]. This is borne out by nucleosome mapping experiments that define potential NURF targets at enhancers, core promoters and insulators [11]. In addition, *Drosophila* mutants lacking NURF subunits exhibit striking disruption of male polytene X chromosomes [12], suggesting that NURF may also function to organize extensive regions of chromatin.

While chromatin remodeling complexes based on ISWI-type catalytic subunits are present in most eukaryotes, NURF appears to be an innovation of the Bilateria. The scaffold for NURF is provided by a highly conserved large subunit [in humans bromodomain and PHD finger transcription factor (BPTF); in *Drosophila* NURF301], which is unique to NURF [13, 14]. The other subunits of NURF are present in multiple chromatin remodeling complexes, indicating that specific functions of NURF, such as targeted recruitment or preferential activity on nucleosomal substrates, are conferred by the BPTF/NURF301 subunit. Consistent with this, BPTF/NURF301 has domains that have the potential to recognize specific HPTMs, including a PHD (plant homeo domain) finger that binds histone H3 lysine 4 trimethylated (H3K4me3) [15] and a C-terminal bromodomain that binds histone H4 lysine 16 acetylated (H4K16Ac) [16]. It has been proposed that binding of H3K4me3 and H4K16Ac by the large subunit controls NURF activity by targeting NURF to specific nucleosomes [16, 17]. However, binding of NURF reader domains to single modifications is weak, making it unlikely that binding single HPTMs is sufficient to mediate selective targeting or tethering *in vivo*. Recognition of multiple adjacent HPTMs by combinations of these reader domains may offer one way to increase binding affinities of NURF for modified nucleosomes and so allow selective recruitment.

Alternatively, it has been proposed weak binding to HPTMs, while insufficient to enable recruitment, may still enable localized focusing of reader complex activity through allosteric inhibition or stimulation of the core enzymatic subunits of chromatin-modifying complexes. Such a sensing function has been proposed for the Polycomb repressive complex 2, where histone methylation activity can be stimulated and inhibited by distinct sets of HPTMs that are detected via the EED or RBBP4 subunits (reviewed in [18]). In the case of NURF, it has been previously observed that histone tails allosterically modulate the ATPase activity of the ISWI catalytic subunit [19], and that HPTMs influence such tail stimulation [20]. In addition, HPTMs can affect interactions of the NURF-55 subunit of NURF with the histone H3 tail [21]. As such, it is formally possible that HPTMs act, not by directly recruiting NURF to nucleosomes to be remodelled, but rather through reader domains sensing HPTMs, to locally stimulate or inhibit the catalytic activity of diffusely distributed NURF complexes.

To discriminate the relative contributions of such tethering or sensing mechanisms to NURF targeting, we sought first to define the full HPTM-recognition specificity of NURF. We concentrated on the large BPTF/NURF301 selective subunit and used modified histone peptide arrays with the objective of identifying individual modifications or combinations of HPTMs that can programme high-affinity binding by BPTF/NURF301 chromatin-interacting domains. We have then used both genomics and single-molecule imaging approaches to discriminate functional roles of these in recruitment and/or regulation of NURF activity. Our data indicate that BPTF/NURF301 binds not to single histone modifications, but rather a battery of HPTMs that decorate the H3 and H4 tails on the +1 nucleosome on active genes. This includes high-affinity recognition of triply modified H3K4me3K9AcS10p by the BPTF/NURF301 PHD2 domain utilizing a novel binding-pocket. We propose this combinatorial recognition of multiple HPTMs is sufficient to cumulatively discriminate and direct NURF recruitment and thus activity to target nucleosomes that include the +1 nucleosome of active genes. In turn NURF orchestrates nucleosome downstream of the +1 nucleosome on active genes to regulate transcription.

## Materials and methods

### Genetics and *Drosophila* strains

The following fly strains were used in this study:

isogenic *w^1118^* described in [11]

*Nurf301^2^* described in [11]

w^1118^; Gcn5^E333st^ P{w[+mW.hs]=FRT(w[hs])}2A e^1^/TM3, P{ActGFP}JMR2, Ser^1^ (RRID:BDSC_9333) described in [22]

y^1^ w*; Df(3L)sex204/TM6C, Sb^1^ Tb^1^ (RRID:BDSC_9337)

*JIL-1^Z2^* [23]

y^1^, w, M{RFP[3xP3.PB] GFP[E.3xP3]=vas-int.Dm}ZH-2A; M{3xP3-RFP.attP}ZH-86Fb (RRID:BDSC_24749)

Unless stated, flies were raised at 25°C and raised on dextrose, wheat flour, yeast, agar media [24].

### Cell lines

*Drosophila* Schneider 2 (S2) cells were obtained from Invitrogen (RRID: CVCL_Z232). S2 cells were cultured at 25°C in protein-free medium containing L-glutamine (Insect-express – Lonza), supplemented with 10% Fetal Bovine Serum (FBS), 100U Penicillin-Streptomycin (Sigma).

### GST fusion protein overexpression and purification

*Escherichia coli* strain BL21 CodonPlus RIL (Agilent) was used as the host for GST-fusion protein overexpression. Protein expression was induced by adding isopropyl β-D-1-thiogalactopyranoside (IPTG, Promega) to 0.5 mM, and the cells were incubated for two and a half hours at 32°C in a shaking incubator at 230 rpm. Cells were harvested by centrifugation at 7900 × *g* for 5 min in an Avanti J20XP centrifuge (JLA10.5 rotor). Harvested cells were washed once with 1× phosphate buffered saline (PBS), and the cell pellet was frozen at −80°C until needed. Cell pellets were resuspended in resuspension buffer (12 mM Hepes at pH 7.9, 12.5 mM MgCl_2_, 2 mM ethylenediaminetetraacetic acid (EDTA), 5 mM Dithiothreitol (DTT), 20% glycerol, 0.1% NP-40). Cells were lysed by sonication on ice (30% power, for 1 min, 10 repeats) using a Vibra-Cell VC130 sonicator (Sonics & Materials). The lysate was cleared by centrifugation twice at 27 000 × *g* for 30 min at 4°C in an Avanti J20XP centrifuge (JA25.5 rotor). The cleared lysate was applied to 0.5 ml of Glutathione Sepharose 4B beads (Amersham) in a Poly-Prep Chromatography Column (Bio-Rad). The column was washed with wash buffer (12 mM Hepes pH 7.9, 12.5 mM MgCl_2_, 2 mM EDTA, 5 mM DTT, 0.15 M KCl, 20% glycerol, and 0.1% NP-40). Bound proteins were then eluted in elution buffer (12 mM Hepes pH 7.9, 12.5 mM MgCl_2_, 2 mM EDTA, 5 mM DTT, 20% glycerol, 0.1% NP-40, 0.15 M KCl, 10 mM glutathione). For NMR, ^15^N-labelled human PHD2 was subjected to on-resin cleavage using PreScission protease (Cytiva) and purified as described [25].

### Histone modified peptide library array analysis

MODified histone H3, H4, H2A, and H2B peptide array (Active Motif) assays were conducted with purified GST-conjugated Bromo, PHD, PHD1, and PHD2 domain proteins, respectively. The proteins were dialysed in Slide-A-Lyzer Dialysis Cassettes (10K MWCO, Thermo Fisher Scientific) in binding buffer (50 mM Tris-Cl, pH 7.5, 150 mM NaCl, 0.1% NP-40) to remove the glutathione. The protein concentrations were determined before they were applied to the peptide arrays. The array was blocked with blocking buffer (50 mM Tris-Cl, pH 7.5, 150 mM NaCl, 0.1% NP-40, 20% FBS, 5 mg/ml biotin) containing protease inhibitor cocktail (one Tablet per 50 ml, Roche) for 5 min at room temperature. The array was washed twice with binding buffer containing 20% FBS for 5 min at room temperature. Each domain protein (concentration range: 1–2 mg/ml) was then introduced to the array, and the binding reaction was performed with binding buffer in a hybridization gasket slide kit overnight at 4°C on a rotator in a microarray Hybridization chamber (Agilent Technologies). Subsequently, the chip was washed with wash buffer (50 mM Tris-Cl, pH 7.5, 150 mM NaCl, 0.1% NP-40) three times for 5 min. To visualize protein-bound peptide spots, the primary antibody reaction was performed with HRP-conjugated rabbit anti-GST antibody (1:5000 dilution), followed by a secondary antibody reaction with IRDye 800 CW rabbit anti-HRP antibody diluted in Chemi IR diluent (1 µg/ml, LI-COR Biosciences) for 1 h at room temperature in the dark. The array was washed three times as described earlier, rinsed with PBS, and spun down briefly in a centrifuge to remove residual buffer on the array. The protein-bound peptide spots were visualized by direct exposure to 800 nm channel of Odyssey scanner (with Odyssey V3.0 programme, LI-COR Biosciences). *n* = 4, as two slides were used for each GST-reader domain fusion, and each slide contains a duplicate array.

### Histone peptide pull-downs

Peptide pull-down was carried out to confirm the peptide array data, as described in [15]. Standard reactions contained 1 µg of biotinylated peptide and 5 µg of purified GST-conjugated protein (Bromo, PHD, PHD1, and PHD2 domain) with 300 µl of binding buffer (50 mM Tris-Cl at pH 7.5, 150 mM NaCl, 0.1% NP-40). However, the amount of peptide in the reaction was varied from 0.1 to 50 µg, depending on the binding affinity of the domain protein to the peptide. Binding reactions were incubated overnight at 4°C on a rotator. Streptavidin Plus UltraLink resin (Thermo Fisher Scientific) was equilibrated by washing with binding buffer four times, and 30 µl of 50% bead slurry was added to each binding reaction. Beads were allowed to bind to biotinylated peptides for 2 h at 4°C on a rotator. Unbound protein was removed by washing with binding buffer four times for 5 min at 4°C. Bound GST-fusion proteins were resolved on 10% Tris-Glycine sodium dodecyl sulphate-polyacrylamide gel electrophoresis (SDS–PAGE) gels using a XCell SureLock Mini-Cell Electrophoresis System (Invitrogen), followed by transfer to PVDF membrane (Bio-Rad) in ice-cold Tris-glycine transfer buffer (12 mM Tris, 96 mM glycine) with 20% methanol at 30 V for 2 h. Membranes were blocked overnight at 4°C in blocking buffer [TBST (50 mM Tris-Cl, pH 7.4, 150 mM NaCl, 0.1% Tween 20) containing 5% dried skimmed milk powder (Marvel)]. Antibody incubation was carried out with HRP-conjugated rabbit anti-GST antibody (1:5000, Novus Biologicals) in blocking buffer for 3 h at room temperature, washed three times for 10 min each in 20 ml TBST buffer, and signal detected using SuperSignal West Pico Chemiluminescent Substrate (Thermo Fisher Scientific). Protein-bound spots were visualized by direct exposure to Hyperfilm ECL (Amersham). Peptides used in these experiments and competition assays are indicated in Table 1. *n *≥ 3, as at least three pull-down experiments were performed for each GST-reader domain fusion.

**Table 1.** Histone tail peptides used for NURF reader binding studies

Biotinylated-Histone H3(1-21)	ARTKQTARKSTGGKAPRKQLAGGK-Biotin	Millipore #12-403
Histone H3(1-21)K4me3	ARTKme3QTARKSTGGKAPRKQLA	Anaspec #64194
Biotinylated-Histone H3(1-21)K4me3	ARTKme3QTARKSTGGKAPRKQLAGGK-Biotin	Millipore #12-564
Biotinylated-Histone H3(1-21)T3pK4me3	ARTpKme3QTARKSTGGKAPRKQLAGGK-Biotin	Cambridge Peptides Custom peptide
Biotinylated-Histone H3(1-21)K4me3K9Ac	ARTKme3QTARKAcSTGGKAPRKQLAGGK-Biotin	Anaspec #64189
Biotinylated-Histone H3(1-21)K4me3K9AcS10p	ARTKme3QTARKAcSpTGGKAPRKQLAGGK-Biotin	Anaspec #65423
Biotinylated-Histone H3(21-44)	ATKAARKSAPATGGVKKPHRYRPGGGK-Biotin	Anaspec #64641
Biotinylated-Histone H3(21-44)K36me2	ATKAARKSAPATGGVKme2KPHRYRPGGGK-Biotin	Anaspec #64442
Biotinylated-Histone H3(21-44)K36me3	ATKAARKSAPATGGVKme3KPHRYRPGGGK-Biotin	Anaspec #64442
Biotinylated-Histone H3(21-44)K36Ac	ATKAARKSAPATGGVKAcKPHRYRPGGGK-Biotin	Anaspec #64936
Biotinylated-Histone H4(1-21)	SGRGKGGKGLGKGGAKRHRKVGSGSK-Biotin	Millipore #12-405
Biotinylated-Histone H4(1-21)K16Ac	SGRGKGGKGLGKGGAKAcRHRKVGSGSK-Biotin	US Biological #H5110-15Q1
Biotinylated-Histone H4(1-25)K16Ac	SGRGKGGKGLGKGGAKAcRHRKVLRDNGSGSK-Biotin	Anaspec #65209
Biotinylated-Histone H4(1-25)K5AcK8AcK12AcK16Ac	SGRGKAcGGKAcGLGKAcGGAKAcRHRKVLRDNGSGSK-Biotin	Anaspec #65248
FAM-H3(1-20)	ARTKQTARKSTGGKAPRKQL-GG-K-FAM	CRB 38854
FAM-H3(1-20)K4me3	ARTKme3QTARKSTGGKAPRKQL-GG-K-FAM	CRB 38855
FAM-H3(1-20)T3pK4me3	ARTPKme3QTARKSTGGKAPRKQL-GG-K-FAM	CRB 38856
FAM-H3(1-20)K4me3K9AcS10p	ARTKme3QTARKAcSpTGGKAPRKQL-GG-K-FAM	CRB 38859
FAM-H4(1-23)	Ac-SGRGKGGKGLGKGGAKRHRKVLR-GG-K-FAM	CRB 38860
FAM-H4(1-23)K16Ac	Ac-SGRGKGGKGLGKGGAKAcRHRKVLR-GG-K-FAM	CRB 38861
FAM-H4(1-21)K5AcK8AcK12AcK16Ac	Ac-SGRGKAcGGKAcGLGKAcGGAKAcRHRKV-GG-K-FAM	CRB 38862

### Peptide competition pull-down assay

Peptide competition pull-down assay was conducted to determine the relative affinities of the NURF301 and BPTF PHD2 domains to different HPTMs on the peptides. Each sample contained 0.1 µg of biotinylated histone-modified peptide (H3(1–21)K4me3, H3(1–21)K4me3K9ac, H3(1–21)K4me3K9acS10p; see Table 1) and 5 µg of purified GST-conjugated PHD2 domain protein with 300 µl of binding buffer and was incubated overnight at 4°C on a rotator. Streptavidin Plus UltraLink resins were equilibrated by washing with binding buffer four times, and 30 µl of 50% bead slurry was added to each sample. The streptavidin resins and biotinylated peptide–protein complexes were allowed to bind to each other for 2 h at 4°C on a rotator. Unbound proteins were removed by washing with binding buffer four times for 5 min at 4°C. Competitor H3(1–21)K4me3 unbiotinylated competitor peptide at 0-, 100-, 250-, and 500-fold molar excess were then added to each sample and incubated for 1 h at room temperature. Unbound peptides were washed with binding buffer four times for 5 min at room temperature, followed by SDS–PAGE and western blotting as described above. *n *≥ 3, as at least three pull-down experiments were performed for each GST-reader domain fusion.

### Fluorescence polarization assay

C-terminal 5-FAM-labelled histone peptides were synthesized and purified by Cambridge Research Biochemicals (CRB) and described in Table 1. Fluorescence anisotropy binding assays were performed in 15 μl with 40 nM fluorescently labelled peptide with increasing concentrations of either BPTF PHD2 or NURF301 bromodomain in binding buffer (50 mM Tris-Cl at pH 7.5, 150 mM NaCl, 0.1% NP-40). Assays were performed in triplicate in low-flange 384-well plates (Corning) and read using a PHERAstar FSX multi-mode reader (BMG Labtech) with an excitation wavelength of 485 nm and an emission wavelength of 520 nm, 50 flashes per well. Values of fluorescence polarization (FP) were converted to anisotropy (A) using the equation *A* = 2*P*/(3 − *P*). Data were fitted in MATLAB using the equation *A* = *A*_f_ + (((*A*_b_ − *A*_f_)*[protein])/(*K*_d_+[protein])). For FP competition assay, binding reactions were performed as above, except 5-FAM-labelled histone H3K4me3 peptide was used at 40 nM, BPTF PHD2 domain at 2 μM, and increasing concentrations of non-fluorescent competitor histone peptides (H3, H3K4me3, H3K4me3K9Ac, H3K4me3K9AcS10p, and H3T3pK4me3) were added. FP was read as earlier and converted to anisotropy values. Calculated data were fitted using GraphPad Prism. *n* = 3, with three determinations performed for every data point.

### NMR analysis

To purify ^15^N-labelled human BPTF PHD2 domain, overnight cultures of *E. coli* strain BL21 CodonPlus RIL (Agilent) expressing BPTF PHD2-GST-fusion protein were seeded into 8 l minimal M9 minimal media supplemented with 0.1% ^15^N-labelled NH_4_Cl (Isotec, 299251) as a nitrogen source and grown to 0.4 OD_600_. Protein expression was induced by addition of (IPTG, Promega) to 0.5 mM and cultures incubated overnight at 18°C in a shaking incubator at 230 rpm. Cells were harvested and GST-PHD2 protein purified as described earlier. ^15^N-labelled human BPTF PHD2 domain was liberated by on-resin cleavage using PreScission protease (Cytiva) and purified as described [25]. Samples were concentrated to 0.4 mM in 50 mM KCl, 20 mM Na/K phosphate pH 7.5, and 5 mM dithiothreitol containing 10% D_2_O (Sigma, 617385) prior to use.

All NMR experiments were acquired on a Bruker 14.1 Tesla Avance III spectrometer equipped with a TCI 5 mm z-PFG cryogenic probe. For the ^1^H-^15^N-HSQC spectra, the Bruker pulse sequence, hsqcetf3gpsi was used. Briefly, in the ^1^H dimension, the carrier was set to the water resonance with a spectral width of 16 ppm and 2K points. In the ^15^N indirect dimension, the carrier was set to 120 ppm with a spectral width of 28 ppm and 512 increments. With a recycle time of 1.2 s and two scans per FID, each spectrum took ~40 min. The assignments of the unbound PHD2 protein in the ^1^H-^15^N-HSQC were obtained from described [25]. The spectra of PHD2 with H3K4me3 peptide or H3K4me3K9AcS10p peptide were obtained by titrating peptide aliquots from a 10 mM peptide stock solution to achieve step increases in concentration 0, 0.08, 0.16, 0.24, 0.32, and 0.4 mM. Peptides used were: histone H3(1–21)K4me3 (Anaspec, 64194) and H3(1–21)K4me3K9AcS10p (Anaspec, 65422).

### Native nucleosome preparation and pull-down

Native nucleosomes were prepared from *Drosophila* S2 cells using a modification of the procedure of Wysocka and colleagues [26]. Briefly, 20 × 10^7^ cells were collected, washed once with 1× PBS, and then resuspended at 4 × 10^7^ cells/ml in buffer A [10 mM HEPES (pH 7.9), 10 mM KCl, 1.5 mM MgCl2, 0.34 M sucrose, 10% glycerol, 1 mM dithiothreitol (Sigma), containing Complete protease inhibitor cocktail (Roche)]. Triton X-100 was added to 0.1% final concentration, and the cells incubated on ice for 8 min to lyse. Nuclei were pelleted by centrifugation at 1300 × *g* for 5 min at 4°C, washed once in buffer A, then lysed in buffer B [3 mM EDTA, 0.2 mM EGTA, 1 mM dithiothreitol (Sigma), with Complete protease inhibitor cocktail (Roche)] on ice for 30 min. Pelleted material was precipitated by centrifugation at 1700 × *g* for 5 min at 4°C and resuspended in buffer C (10 mM Tris, 10 mM KCl, and 1 mM CaCl_2_). Cells were treated with MNase (Worthington) at 30U/10^7^ starting cells for 2.5 min at 37°C. Digestions were stopped by addition of EGTA to 1 mM final concentration. Samples were centrifuged at 17 000 × *g* for 5 min at 4°C and supernatant harvested. 1/10 volume was reserved for input and the remaining material used for IP with anti-NURF301 coated beads. Prior to pull-down 50 µl protein G-coated magnetic Dynabeads M-280 (Invitrogen) were washed four times for 5 min on a rotator at RT using 1 ml 1× PBS containing 5 mg/ml BSA (Invitrogen) and protease inhibitors (Complete, Roche). After the final wash, beads were then resuspended in 500 µl 1× PBS containing BSA. One hundred microlitres of anti-NURF301 antisera were added to beads and incubated overnight at 4°C. Beads were washed four times for 5 min on a rotator at RT using 1 ml 1× PBS containing 5 mg/ml BSA (Invitrogen). Soluble chromatin following MNase digestion was added directly to washed beads and incubated at 4°C for 2.5 h. Beads were washed four times for 5 min on a rotator at RT using 1 ml 1× PBS containing 5 mg/ml BSA (Invitrogen) and protease inhibitors (Complete, Roche). Input chromatin and beads were made to 1× with Novex Tris-Glycine SDS Sample Buffer (Invitrogen). Bound proteins were eluted by boiling in 1× SDS–PAGE sample buffer. Input and IP samples were normalized using pan-H3 antibodies, and western blots performed using the following antibodies at the indicated dilutions: rabbit anti-histone H3 (Abcam ab1791, 1:2000); rabbit anti-histone H3K4me3 (gift of Bryan Turner,1:1000); rabbit anti-histone H3T3p (Millipore 07-424,1:5000); rabbit anti-histone H3K9AcS10p (Abcam ab12181,1:5000); rabbit anti-histone H4K16Ac (Serotec AHP417,1:2000), and H3K9Ac (gift of Bryan Turner,1:5000). *n* = 3, with at least three western blots performed per histone modification.

### *galK* recombineering and gap repair for *Nurf301* tagging and mutation

BAC clones *CHORI-CH321-85D11* and *CHORI-CH322-169D05* (BACPAC Resources Center), which contain the *Nurf301* (*E(bx)*) genomic interval, were used for tagging by *galK*-mediated recombineering [27] as described in Supplementary Methods, Supplementary Table S1, and Supplementary Figs S6–S8. BAC clones were transferred to the recombinogenic *E. coli* strain *SW102*, and *galK* coding sequence inserted into *Nurf301* coding sequence to replace the termination codons of either the full-length *Nurf301-A* or short *Nurf301-C* transcripts. Primers used for amplifying *galK* with *Nurf301* 400–500 bp homology arms are described in Supplementary Methods. Electrocompetent cells of *E. coli SW102* containing either CH321/CH322 BAC construct were prepared as described [27] from both non-heat-shocked and heat-shocked cells, which induce the expression of the recombineering functions. Uninduced and induced competent cells were transformed with 300 ng of *galK* template DNA (*Nurf301* full A/B and C isoforms) with 500 bp arms by electroporation, resuspended with 1 ml M9 medium, and serial dilutions plated onto *galK*-positive selection plates [M63 medium with 15 g/l agar (BD Biosciences), 0.2% D-galactose (Sigma), 1 mg/l D-biotin (Sigma), 45 mg/l L-leucine (Sigma), and 12.5 μg/ml chloramphenicol (Sigma)]. Plates were incubated at 30°C, and eight *galK*+ colonies of from each plate were streaked on Gal indicator plates [MacConkey agar (BD Biosciences), 1% D-galactose (Sigma), and 12.5 μg/ml chloramphenicol (Sigma)] to identify *galK*- contaminating ‘hitchhikers’.

Substitution templates were then prepared by PCR, which contained identical homology arms used for *galK* insertion flanking coding sequences for either the GS-TAP tag (*Streptococcus* protein G and streptavidin-binding peptide [28]), EYFP (Clontech #6006-1), or the HaloTag [29]. Sequence-verified *galK*+ SW102 clones were used to generate uninduced and induced competent cells, which were transformed with 300 ng of tag template DNA flanked by 500 bp homology arms. Following recovery, transformed cells were washed in M9 medium and serial dilutions plated onto *galK* counter-selection plates [M63 medium with 15 g/l agar (BD Biosciences), 0.2% glycerol (Sigma), 1 mg/ D-biotin (Sigma), 45 mg/l L-leucine (Sigma), 0.2% 2-deoxy-D-galactose (DOG, Merck), and (12.5 μg/ml chloramphenicol (Sigma)]. Plates were incubated at 30°C, and DOG-resistant colonies in which *galK* was replaced by tags were identified by colony PCR using primers in the tag and flanking *Nurf301* genomic sequence. Tagged BACs were purified by plasmid DNA miniprep and transformed into *E. coli* strain EPI300 (Epicentre) by electroporation (see 2.2.6.4) for large-scale purification as described in Supplementary Methods. Mutations in the NURF301 PHD2 domain were engineered in NURF301-A YFP- and HaloTag tagged BACs using an identical *galK*-mediated recombineering approach, with the exception that recombineering substituted the wild-type (WT) PHD2 sequence with repair templates containing W32A (W2519A) and R36E E46K (R2523E and E2533K) mutations.

To generate tagged NURF301 transgenic lines, the φC31 integrase system was utilized [30]. DNA was injected into embryos of the strain *y^1^, w, M{RFP[3xP3.PB] GFP[E.3xP3]=vas-int.Dm}ZH-2A; M{3xP3-RFP.attP}ZH-86Fb*, which carries an *attP* docking site, at 86F on chromosome 3R and which expresses φC31 integrase in the germline and red-eyed transgenic founders selected.

### Immunostaining of *Drosophila* polytene chromosomes

*Drosophila* larvae were raised in non-crowded conditions (~15 female flies were allowed to lay eggs in a vial containing medium and transferred to a new vial every 2 days). To get optimal polytene chromosome morphology, larvae were raised at 22°C, and uncrowded culturing of larvae was essential. Wandering-stage third-instar larvae were washed in water and transferred to Brower’s fixation buffer [31] (0.15 M Pipes, 3 mM MgSO_4_, 1.5 mM EGTA, and 1.5% NP40 at pH 6.9) containing 2% formaldehyde, and salivary glands were directly dissected in this solution. The glands were fixed for 2–3 min and transferred to PBT (PBS containing 0.1% Triton X-100) for another 2–3 min. They were moved into 50% glycerol and soaked for 5 min. The glands were transferred to 13 µl 50% glycerol on a 22 mm × 22 mm Sigmacote-treated coverslip, broken down to small pieces using a tungsten needle, and squashed onto poly-L-lysine-coated microscope slides. Slides were directly submerged in liquid nitrogen for a few seconds to freeze, and the coverslip was removed using a razor blade. The frozen slide was quickly submerged in 1× PBS until sufficient slides had been collected for immunostaining. For double-labelling, slides were sequentially stained with primary antibodies. Slides were incubated with first primary antibody in PBTw (1× PBS + 0.1% Tween-20) containing 10% BSA at 4°C overnight. Samples were then washed with PBTw four times for 5–10 min at room temperature. The first secondary antibody reaction was conducted using Cy3-conjugated AffiniPure Fab fragment goat anti-rabbit IgG (Jackson ImmunoResearch, 1:400) in PBTw for 2 h at room temperature in the dark. The samples were washed with PBTw three times for 10 min, then fixed in 1% formaldehyde for 10 min in the dark, followed by three 10-min washes in PBTw. Samples were blocked with unconjugated AffiniPure Fab fragment goat anti-rabbit IgG (Jackson ImmunoResearch, 1:20) in PBTw for 2 h at room temperature in the dark and washed in PBTw as earlier, and the second primary antibody (host rabbit) reaction was performed in PBTw containing 10% BSA overnight at 4°C. The samples were washed with PBTw three times for 10 min on the following day. The second secondary antibody reaction was performed using FITC-conjugated AffiniPure Fab fragment goat anti-rabbit IgG (Jackson ImmunoResearch, 1:400) in PBTw for 2 h at room temperature in the dark. Samples were washed with PBTw as previously, mounted in Vectashield with DAPI (Vecta Laboratories), and examined by confocal microscopy using a Zeiss LSM780 confocal microscope. The following primary antibodies were used at the indicated dilutions: rabbit anti-histone H3K9Ac (1:250, gift of Bryan Turner); rabbit anti-histone H3K9AcS10p (1:250, Abcam, ab12181); rabbit anti-NURF301 [24] (1:25); rabbit anti-protein G (1:500, Novus Biologicals NB600-1086).

### Live imaging of salivary gland polytene chromosomes

Wandering third-instar stage YFP-tagged NURF301-A-expressing transgenic larvae were raised in non-crowded conditions as described earlier, washed from media, and salivary glands dissected in Schneider’s *Drosophila* Medium (Thermo) and immobilized on slides by placing in ~25 μl of Schneider’s *Drosophila* Medium sandwiched between 22 mm × 22 mm cover slips (Menzel-Glaser) under 50 mm × 22 mm cover slip (Menzel-Glaser). Live imaging was performed using a Zeiss LSM 780 AxioObserver.Z1 multiphoton laser scanning confocal microscope fitted with the Pecon incubation system. YFP excitation was achieved using Lasos 25 mW LGN3001 multiline 458, 488, and 514 nm Argon laser, and emission detected using a gallium arsenide phosphide detector. All images were captured utilizing the water-immersion 40× c-achromatic (NA: 1.2) objective lens. To discriminate the contributions of histone H3K9Ac and H3S10p to NURF301 recruitment, YFP-tagged NURF301-A transgenes were recombined onto *Gcn5^E333st^* or *JIL-1^Z2^* mutant chromosomes, and homozygous mutant salivary glands imaged. Images of at least 100 polytene nuclei per genotype from multiple salivary glands were captured. Images were captured using identical capture settings for all experiments. Unbound/bound ratios were calculated for each nucleus by determining signal intensities using a standard regions of interest (ROI) with multiple assay points on polytene bands (for bound) and assay points in nucleoplasm (for unbound). Averaged ratio was calculated from these values for each nucleus and box and whiskers plots generated from at least 100 nuclei per genotype.

### FRAP analysis of salivary gland polytene chromosomes

Salivary glands were dissected and mounted as described earlier. High-resolution images (bidirectional scanning, pixel dwell time of 6.3 μs, 16× averaging) were captured to identify ROI. A 10-scan time-series (1× averaging, 500 ms interval, and pixel dwell time of 0.5 μs) was taken before bleaching to establish ROI baseline fluorescent intensity. Bleaching was performed using a 488 nm laser at 50% power for 10 iterations, and multiple ROIs (both bleached regions and unbleached regions; *n* = 5 per nucleus) were imaged for a further 120 s, using identical parameters as pre-bleach, to monitor fluorescence recovery. Fluorescence recovery after photobleaching (FRAP) analysis was performed using the LSM FRAP Module (Zeiss). Half-life of recovery was calculated for each nucleus, and averaged half-life of recovery determined for each genotype from at least five determinations.

### Hemocyte isolation

Primary hemocytes from third-instar larvae were isolated as described previously [11]. Briefly larvae were ripped in batches of 50 third-instar larvae into HyQ-CCM3 insect medium (Thermo Fisher Scientific) containing protease inhibitors (Complete, Roche). For imaging, cells were pelleted at 300 × *g* for 5 min at room temperature and resuspended in Schneider’s *Drosophila* Medium (Thermo). For transcriptome analysis, cells were pelleted at 300 × *g* for 5 min, washed with ice-cold 1× PBS containing protease inhibitors, and stored as pellets at −80°C until required. For chromatin immunoprecipitation sequencing (ChIP-seq), cells were fixed with 1% formaldehyde in 1× PBS for 15 min at 25°C, pelleted at 300 × *g* for 5 min then washed three times with ice cold 1× PBS containing protease inhibitors and stored as pellets at −80°C until required.

### Single particle tracking of NURF301 variants

Hemocytes were prepared from transgenic larvae expressing NURF301-A or NURF301-C HaloTag fusions as described earlier. Resuspended hemocytes were transferred to an Attofluor cell chamber (Invitrogen) containing #1.5 25 mm round coverglass (Electron Microscopy Sciences, 72225-01). Cells were allowed to settle and attach for 30 min, then the media discarded and replaced with Schneider’s *Drosophila* Medium containing 0.5 nM of JF 549 HaloTag ligand. Hemocytes were incubated at room temperature in the dark for 10 min, washed with 1 ml Schneider’s medium three times, and imaged using a Nikon N-STORM microscope using a 100× oil-immersion TIRF objective to conduct single-particle tracking experiments. Images were captured at 500 ms intervals, 300 frames per movie, and stored as nd2 files. These were converted to TIF format using ImageJ (https://imagej.nih.gov/ij/) and imported to Diatrack [32] for particle tracking. The resultant tracks were converted into MATLAB files so that statistical analysis could be performed. Scripts from the Sojourner particle tracking package (https://github.com/sheng-liu/sojourner) were used in RStudio to analyze the images. First, the createTrackll.R script was run to select the files and import tracks. After that, the combineTrackll.R script was run so that the files are all combined to form one analysis. Then, the linkSkippedFrames.R script was run to link the tracks in which particles may have been skipped in single frames of the original movie. Following that, the dwellTime.R script was run, followed by the fitResidenceTime.R script to generate plots of particle residence time. This step was conducted for cells recorded on different days, and the data were then merged to generate one representative data set for all the cells. We analysed at least 10 000 tracks for each construct to determine average residence time on chromatin.

### Chromatin immunoprecipitation sequencing

Antibody-coated beads were prepared the day before the ChIP. Fifteen microlitres protein G-coated magnetic Dynabeads M-280 (Invitrogen) were used per ChIP sample for pre-immune incubation, 40 µl for each ChIP. Beads were washed four times for 5 min on a rotator at RT using 1 ml 1× PBS containing 5 mg/ml BSA (Invitrogen) and protease inhibitors (Complete, Roche). After the final wash, the beads were then resuspended in 500 µl 1× PBS containing BSA. Two micrograms of ChIP antibody were added to each ChIP bead sample. Both ChIP (+Ab) and pre-immune (−Ab) beads were incubated overnight on a rotator at 4°C. On the day of the ChIP, beads were washed five times in 1 ml 1× PBS containing BSA to remove any unbound antibody and finally resuspended in 50 µl ChIP dilution buffer.

Chromatin for ChIP was either sheared by sonication (*Drosophila* S2 cells) or by MNase digestion (hemocytes). For sonication, either 150 million (NURF301 ChIP) or 10 million cells (HPTM ChIP) were resuspended in 150 µl SDS lysis buffer (1% SDS and 10 mM EDTA) and sonicated for either 12.5 or 20 min, respectively, using a Diagenode Bioruptor sonicator (cycles of 30s on/off, hi power). Cells were centrifuged at 24 000 × *g* for 1 min at 18°C to pellet insoluble material, and supernatant was transferred onto a fresh 1.5 ml LoBind tube (Eppendorf). Samples were then diluted to 1.5 ml using ChIP dilution buffer (16.7 mM Tris-Cl, pH 8.1, 167 mM NaCl, 1.2 mM EDTA, 0.01% SDS, and 1.1% Triton X-100). For MNase digestion, hemocytes corresponding to 1000 *w^1118^* larvae were pooled and MNase-digested as described [11]. MNase reactions were stopped by adding stop buffer (0.1 M Tris, pH 8.5, 0.1 M NaCl, 50 mM EDTA, 1% SDS) and diluting to 1.5 ml with ChIP dilution buffer (16.7 mM Tris-Cl, pH 8.1, 167 mM NaCl, 1.2 mM EDTA, 0.01% SDS, and 1.1% Triton X-100).

From this point, both ChIP protocols followed identical procedure. Aliquots of pre-immune beads were added to the soluble chromatin and incubated at room temperature on a rotator for 15 min. Beads were pelleted using a magnetic rack, and the cleared supernatant was transferred into a fresh LoBind tube. Fifty microlitres of soluble chromatin was removed to be used as input and stored on ice. To the remaining chromatin, the antibody bead slurry was added and incubated for 2.5 h at room temperature on a rotator to form immune complexes. Beads were then washed using in sequence low-salt buffer (20 mM Tris-Cl, pH 8.1, 150 mM NaCl, 2 mM EDTA, 0.1% SDS, 1% Triton X-100), high-salt buffer (20 mM Tris-Cl, pH 8.1, 500 mM NaCl, 2 mM EDTA, 0.1% SDS, and 1% Triton X-100), and LiCl buffer (10 mM Tris-Cl, pH 8.1, 0.25 M LiCl, 1 mM EDTA, 1% IGEPAL-CA630, and 1% deoxycholic acid).

For each wash, beads were incubated at room temperature on a rotator, snap-spun, and pelleted using a magnetic rack for 2 min, with the supernatant removed before proceeding to the next wash. Finally, beads were washed twice in TE buffer (10 mM Tris-Cl, pH 8.0, and 1 mM EDTA), supernatant removed, and ChIP DNA eluted using 75 µl of freshly prepared and filter-sterilized elution buffer (1% SDS, 0.1 M NaHCO_3_). Elution was performed at room temperature on a rotator for 15 min, and the elution repeated. Eluates were pooled, and corresponding inputs diluted to 150 µl using elution buffer; 5 µl of proteinase K (50 mg/ml) and NaCl, Tris, and EDTA added to all samples at a final concentration of 250, 20, and 10 mM, respectively. Samples were incubated overnight at 65°C to digest protein and reverse crosslinks, and DNA purified using 1.8 volumes of Agencourt AMPure XP beads (Beckman Coulter) following the supplier’s protocol. DNA for MNase-seq and ChIP-seq was end-repaired and sequencing libraries prepared using a SOLiD Fragment Library Construction Kit (Life Technologies). ChIP DNA was barcoded using the SOLiD Fragment Library Barcoding Kit Module 1–16. Sequencing libraries were run on a SOLiD 4 Genome Analyzer. The following antibodies were used for ChIP: Rabbit anti-histone H3K4me3 (Millipore #17-614), Rabbit anti-histone H3K9AcS10p (Abcam ab12,1:5000), Rabbit anti-histone H4K16Ac (Serotec AHP417), Rabbit anti-histone H4K16Ac (Millipore #17-10101), H3K9Ac (Gift of Bryan Turner), and Rabbit anti-NURF301 [11]. *n* = 2, with two libraries generated per antibody and cell type.

### CAGE-seq

Total RNA was extracted from hemocytes from 1000 larvae (either *w^1118^* or *Nurf301^2^*) per library using an RNeasy Mini Kit (Qiagen) followed by DNase treatment as described in the manufacturer’s instructions. Cap analysis of gene expression (CAGE)-seq libraries were prepared and sequenced by DNAForm (Riken, Japan) and mapped to the genome using BWA [33], and reads used to determine transcription start site (TSS) locations genome-wide in both WT and *Nurf301* mutant hemocytes. Specifically, a BED file containing all CAGE reads for each genotype was used to determine read number at each genome coordinate using the genomecov utility of BEDTools [34]. Tracks were filtered to define bona fide initiation points in WT by filtering for genome intervals containing >10 mapped reads. Peak value, which defines the TSS in each interval, was then determined using the bigWigSummary utility of kentUtils (https://github.com/ENCODE-DCC/kentUtils). These coordinates were then used to profile flanking initiation in WT and *Nurf301* hemocytes using the Heatmap function of deepTools [35] or for averaged profiling using the sitepro function of the CEAS: *cis-*regulatory element annotation system [36]. Expression analysis was performed by counting CAGE reads in a 150 bp window flanking these called TSSs in both WT and *Nurf301* hemocytes using the coverage utility of BEDTools. Using total number of CAGE reads, normalized count per million values were determined at each TSS in both WT and *Nurf301* mutants. Scatterplots of expression ratios between WT and *Nurf301* were generated using the ggplot2 data visualization package in R. *n* = 2, with two libraries generated per genotype.

### mRNA-seq

Messenger RNA (mRNA) was extracted from hemocytes from 1000 larvae per library using a MACS mRNA Isolation Kit (Miltenyi Biotec), and libraries prepared for sequencing using a SMARTer Stranded RNA-Seq Kit (Clontech) according to the manufacturer’s instructions, with the following modifications: heat fragmentation of RNA was performed for 3 min at 94°C. Libraries were barcoded and then run on an Agilent Bioanalyzer 2100 with HS DNA chip to confirm library size centred on 300 bp. mRNA-seq libraries were sequenced using a commercial sequencing provider (Macrogen, Korea) on an Illumina HiSeq 2500 sequencer. Read quality was assessed using FastQC (http://www.bioinformatics.babraham.ac.uk/projects/fastqc/). Reads were then mapped and expression determined using STAR [37] and RSEM [38]. Normalized counts per million statistics were generated for all expressed genes using RSEM, and scatterplots of expression ratios between WT and *Nurf301* were generated as above. *n* = 2, with two libraries generated per genotype.

## Results

### Peptide array screening defines HPTMs bound by NURF301 reader domains

**Figure 1.**
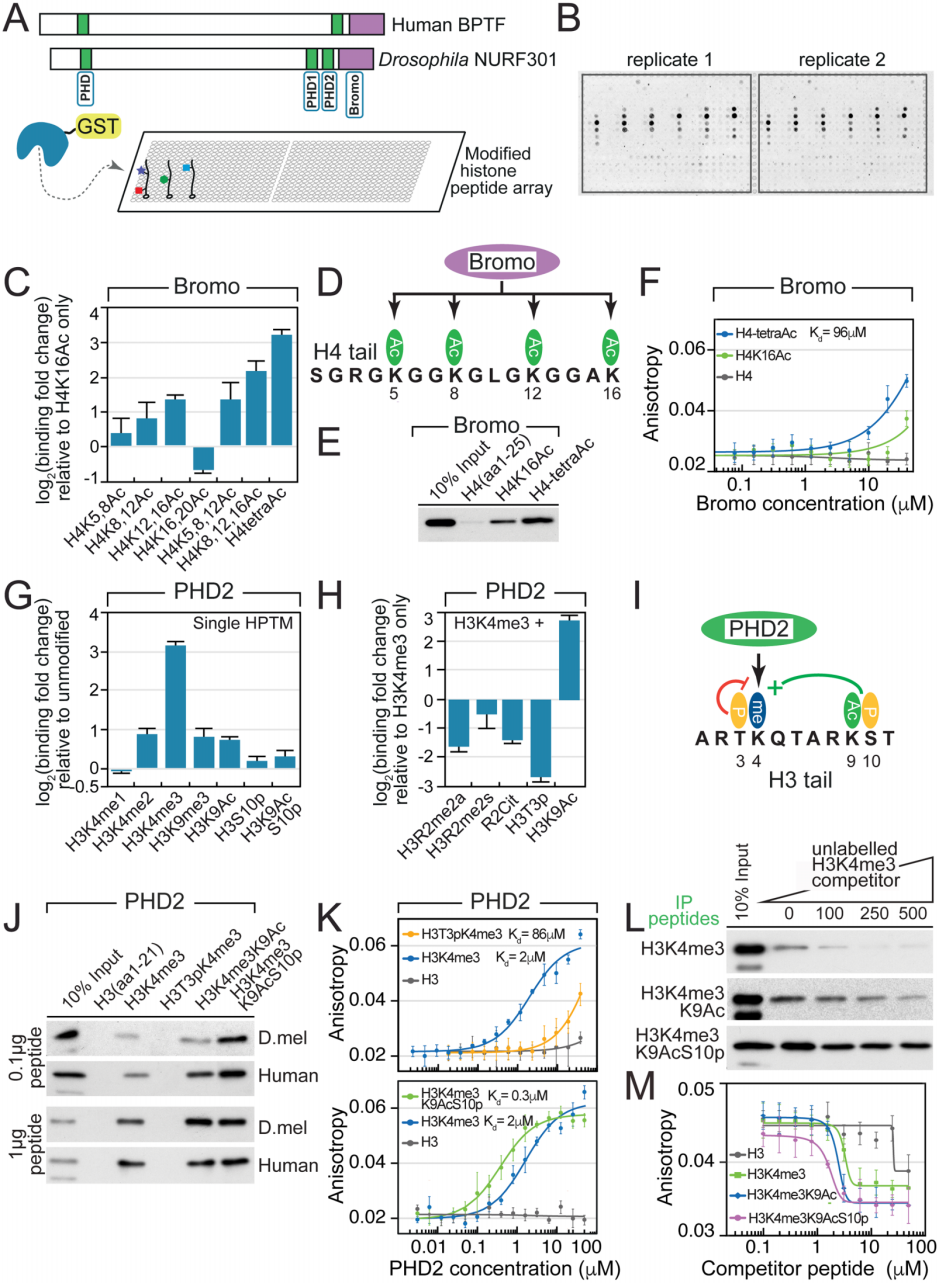
Profiling histone tail recognition by NURF reader domains. (**A**) Overview of histone peptide array assays. Purified GST-tagged NURF301 PHD, PHD1, PHD2, or bromodomain fusion proteins were incubated with modified histone peptide arrays and binding detected by slide scanning. (**B**) Representative peptide array showing reproducible binding of the GST-PHD2 domain. (**C**) Peptide array analysis shows that the bromodomain preferentially binds multiply acetylated H4 tails. (**D**) Schematic overview of rheostat modifications regulating bromodomain binding. (**E**) Histone peptide pull-down assays confirm efficient binding of the NURF301 bromodomain to tetra-acetylated H4 tails. (**F**) FP assays measuring bromodomain binding to H4K16Ac and tetra-acetylated H4 peptides; unmodified H4 serves as control. Data represent mean ± SD of three independent assays. (**G**) Peptide array analysis reveals that the PHD2 domain preferentially binds H3K4me3 modification when present as a single modification. (**H**) H3K4me3 recognition is modulated by neighbouring H3 modifications. (**I**) Schematic overview of rheostat modifications affecting binding of the PHD2 domain. (**J**) Histone peptide pull-down using limiting amounts of biotinylated peptide [0.1 μg peptide (44 pmol) versus 1 μg peptide (440 pmol) with 130 pmol of protein] confirms enhanced binding of human and *Drosophila* NURF PHD2 domain to triply modified H3K4me3K9acS10p peptides. (**K**) FP assay of human BPTF PHD2 to FAM-labelled H3 peptides allows *K*_d_ for modified peptides to be determined. Data represent mean ± SD of three independent assays. (**L**) Histone peptide competition pull-down assays in which binding to biotinylated H3K4me3, H3K4me3K9ac, and H3K4me3K9acS10p peptides is competed by 0–500 molar excess of competitor H3K4me3 peptide demonstrates stable binding to triply modified H3K4me3K9acS10p peptide. (**M**) Peptide competition FP assay was performed with 2 μM human BPTF PHD2, 40 nM FAM-labelled H3K4me3 peptide, and increasing concentrations of unlabelled competitor H3 peptides, revealing stepwise increase in affinity of PHD2 for H3K4me3 < H3K4me3K9Ac < H3K4me3K9AcS10p. Data represent mean ± SD of three independent assays.

Previous studies showed that NURF binds to H3K4me3 and H4K16Ac through the bromodomain and PHD2 finger of the specificity subunit BPTF/NURF301 [15, 16]. To determine if there are additional HPTMs that could be bound by NURF, we examined binding of these domains as well as two additional PHD motifs that occur in BPTF/NURF301 (Fig. 1A) to histone peptide arrays. These arrays contained combinations of 59 HPTMs, including acetylation, methylation, phosphorylation, and citrullination on the H2A, H2B, H3, and H4 N-terminal tails, and contain 384 different combinations of single to quadruple modifications, allowing the effect of combinations of HPTMs on binding to be determined (Fig. 1B).

As expected, the bromodomain showed binding to H4K16Ac peptides but also bound other singly acetylated lysines on histone H4, including H4K5Ac, H4K8Ac, and H4K12Ac (Supplementary Fig. S1E and Fig. 1C). However, binding to di-, tri-, or tetra-acetylated H4 peptides was enhanced, with the tetra-acetylated histone H4 tail [Lys5, Lys8, Lys12, Lys16 (H4tetraAc)] showing strongest binding (Fig. 1C). This was confirmed by peptide pull-down (Fig. 1E) and FP assays (Fig. 1F). Although the NURF301 bromodomain could bind H4K16Ac, binding to the multiply-acetylated H4tetraAc peptide was stronger with a calculated *K*_d_ of 96 μM (Fig. 1F).

While two of the BPTF/NURF301 PHD domains (designated PHD and PHD1 according to [15], Fig. 1A) showed weak binding to modifications of H3K36 (Supplementary Fig. S1B and C), these were not confirmed by peptide pull-down, where no binding could be detected (Supplementary Fig. S2). In contrast, the C-terminal PHD2 domain showed significant binding to peptide spots containing H3K4me3 (Fig. 1G). The PHD2 domain did not bind peptides containing H3K4me1 or H3K4me2, and no significant binding was observed to any other singly modified tails, indicating that PHD2 specifically binds to H3K4me3 (Fig. 1G). However, additional flanking modifications modulated the binding of PHD2 to H3K4me3. These fell into two categories: switches that turned off recognition of H3K4me3 and rheostats that increased binding to H3K4me3 (Fig. 1I). Examples of switches included phosphorylation of Thr3 (H3T3p), which reduced binding to 12% of that observed with H3K4me3 alone, and dimethylation of Arg2, which had weaker inhibitory effects, reducing PHD2 binding to 30% (H3R2me2aK4me3) and 60% (H3R2me2sK4me3) relative to H3K4me3 alone (Fig. 1H). Switch activity of H3T3p was confirmed by peptide pull-down and FP assay, with H3T3p significantly inhibiting binding of the PHD2 domains of both human BPTF and fly NURF301 (Fig. 1J and K) to H3K4me3 peptides. This inhibition was consistent with previous data on mitotic chromosomes where immunolabeling for H3T3p or H3T3pK4me3 and either BPTF or NURF301 showed no overlap [39].

### The BPTF/NURF301 PHD2 domain recognizes triply modified histone H3K4me3K9AcS10p tail

In contrast, acetylation of histone H3 Lys9 (H3K9Ac) acted as a rheostat, increasing binding of the PHD2 domain to H3K4me3-modified peptides approximately six-fold (Fig. 1H), with enhancement confirmed by histone peptide pull-down assay (Fig. 1J) and histone peptide competition binding experiments (Fig. 1L and M). On active promoters, H3K9Ac is frequently accompanied by phosphorylation of Ser10 (H3S10p) [40]. As phosphorylation of H3T3 inhibited PHD2 binding to H3K4me3, we tested whether H3S10p also antagonized binding to H3K4me3K9Ac peptides. In fact, we observed further stepwise enhancement of PHD2 binding following H3S10 phosphorylation (Fig. 1J). This was confirmed both by peptide pull-down assays (Fig. 1J) and FP assays, where triple modification (H3K4me3K9AcS10p) decreased the *K*_d_ for BPTF PHD2 from 2 μM (H3K4me3 peptide) to 300 nM (Fig. 1K). These results were verified by competition experiments, in which binding of fly or human PHD2 domains to either biotinylated H3K4me3, H3K4me3K9Ac, or H3K4me3K9AcS10p peptides was competed using increasing concentrations of competitor non-biotinylated H3K4me3 peptide (Fig. 1L). We observed a sequential increase in PHD2 binding affinity after each modification. Thus, binding to biotinylated H3K4me3 peptide was readily competed by excess unlabelled H3K4me3 peptide, binding to H3K4me3K9Ac was resistant to 100–250-fold molar excess, and binding to H3K4me3K9AcS10p peptides resistant to 500-fold molar excess of H3K4me3 competitor. Similarly, peptide competition FP assay likewise demonstrated an ordered increase in PHD2 binding affinity with the addition of each modification (Fig. 1M).

### Two binding surfaces on BPTF/NURF301 PHD2 for H3K4me3K9AcS10p

**Figure 2.**
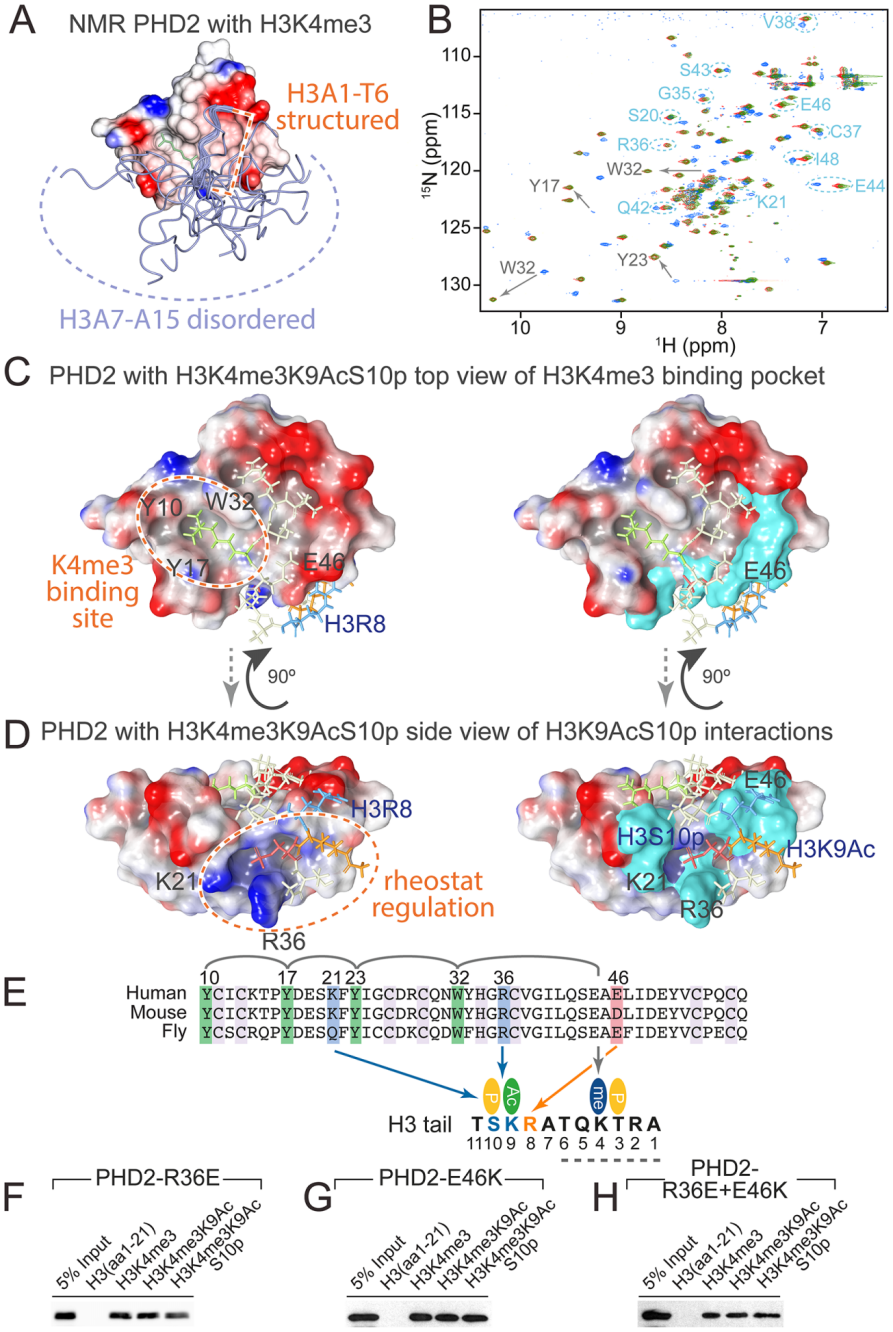
Structural characterization of the H3K9AcS10p interaction surface on the human BPTF PHD2 domain. (**A**) NMR structure of BPTF PHD bound to singly modified H3K4me3, demonstrating that H3 residues A7–A15 adopt multiple conformations when binding singly modified H3K4me3 peptides (PDB ID: 2FUU). (**B**) Superposition of BPTF PHD2 ^1^H,^15^N-HSQC NMR spectra of amide resonances without H3 peptide (blue), or with singly modified H3K4me3 peptide (green) or triply modified H3K4me3K9AcS10p peptide (red), identifies residues that differentially interact with triply modified H3K4me3K9AcS10p. (**C**) Top and (**D**) side view of the PHD2 domain with amino acid residues that show differences between H3K4me3 and H3K4me3K9AcS10p binding modelled in cyan on the existing crystal structure of the BPTF PHD2 domain in complex with H3K4me3 (PDB 2F6J). The histone tail peptide is indicated in grey with H3K4me3 shown in green, H3R8 shown in blue, H3K9Ac shown in orange, and H3S10p shown in red. Charged residues that are predicted to interact with H3 tail peptide in the presence of H3K9AcS10p are indicated. (**E**) Amino acid sequence alignment of the PHD2 domains from mouse and human BPTF and fly NURF301. Residues that constitute the H3K4me3-recognizing hydrophobic cage are indicated in green. Conserved residues R36 and E46 are predicted to make contacts with the H3K9AcS10p and H3R8, respectively. (**F**–**H**) Histone peptide pull-down assay confirms that R36 and E46 are necessary for selective H3K4me3K9acS10p recognition. Mutation of residues does not affect H3K4me3 recognition, but blocks enhanced binding of H3K4me3K9acS10p.

To define the structural basis for the enhanced recognition of triply modified H3K4me3K9AcS10p tail by PHD2, we exploited existing X-ray crystal and NMR structures of the PHD2 domain of human BPTF in complex with singly modified H3K4me3 peptide [25]. In both these structures, although the H3 N-terminal six amino acids flanking the H3K4me3 modification are ordered and resolved, the distal regions of the H3 tail that encompass the H3K9 and H3S10 residues are disordered and assumed multiple conformations in the NMR structure (Fig. 2A). We speculated that the presence of H3K9AcS10p modification, in addition to H3K4me3, would stabilize the H3 tail through binding to additional surfaces on the PHD2 domain.

To identify these interacting residues, ^15^N-labelled human BPTF PHD2 domain was over-expressed and purified as described [25], and ^1^H-^15^N-HSQC spectra determined for the PHD2 domain alone or in complex with singly modified H3K4me3 or triply modified H3K4me3K9AcS10p peptides (Fig. 2B). Comparison of spectra allowed amino acid residues that interact with K9AcS10p to be identified and modelled onto the existing structure of the BPTF PHD2 domain (Fig. 2C and D, shown in cyan). This identified a cluster of residues, including K21, R36, and E46, that creates a second binding region on the PHD2 domain for H3K9AcS10p, distinct from the hydrophobic cage responsible for H3K4me3 recognition. By modelling the trajectory of the H3 tail from H3T6 to H3S10, we defined putative interactions between H3 tail modifications and amino acids on PHD2. This model indicated that, when H3K9 is acetylated and H3S10 is phosphorylated, binding to H3K4me3 can be further stabilized by charge interactions between PHD2 residues K21, R36, and H3K9AcS10p, and between E46 of the PHD2 domain and unmodified H3R8 (Fig. 2). To verify that these residues were required for enhancement of H3K4me3 binding by H3K9AcS10p, we generated charge-switch mutations in the PHD2 domain, introducing both the single substitutions R36E and E46K, and producing the corresponding double mutant, R36E + E46K. Histone peptide pull-down assay demonstrates that mutations in the rheostat regulation domain did not block H3K4me3 binding but were able to prevent enhanced binding to H3K4me3K9Ac and H3K4me3K9AcS10p peptides (Fig. 2F–H).

### NURF associates with multiply modified nucleosomes

**Figure 3.**
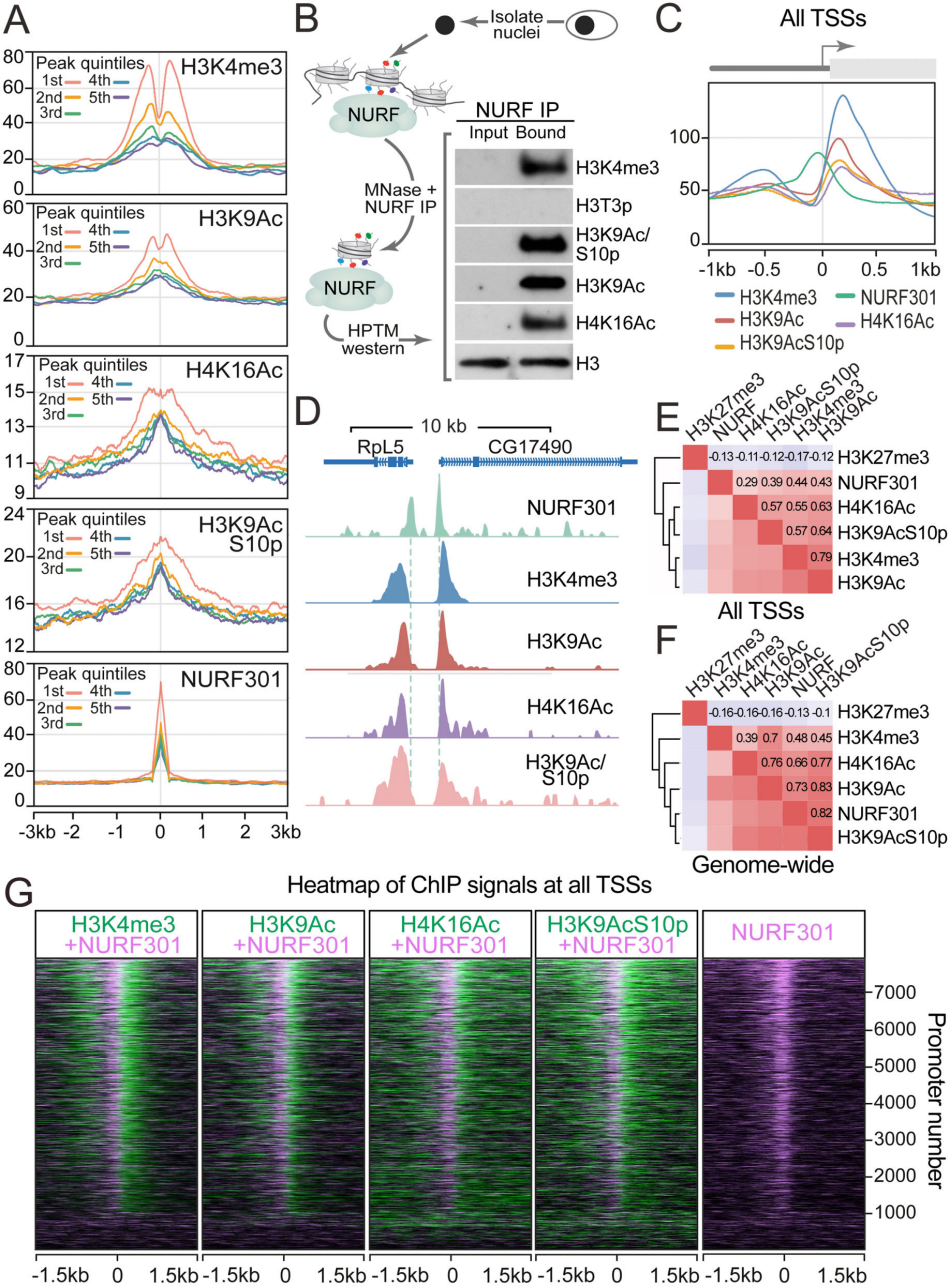
NURF colocalizes with HPTMs *in vivo* that decorate the +1 nucleosome. (**A**) NURF301 ChIP peaks were ordered according to strength of associated ChIP signal to identify 5456 robust sites, which were further divided into quintiles from highest (first) to lowest (fifth) signal. Normalized H3K4me3, H3K9Ac, H3K9AcS10p, H4K16Ac, and NURF301 tag density were determined relative to NURF301 peaks, revealing HPTMs flank NURF301 peaks and correlate with strength of NURF301 ChIP signal. (**B**) Immunoprecipitation using anti-NURF301 antibodies of native mononucleosomes isolated from S2 cells reveals that nucleosomes associated with NURF are enriched in H3K4me3, H3K9Ac, H3K9AcS10p, and H4K16Ac but lack H3T3p. Pan H3 western provides control. (**C**) Normalized NURF301, H3K4me3, H3K9Ac, H3K9AcS10p, and H4K16Ac tag density relative to TSSs. (**D**) ChIP-seq analysis shows NURF301 peaks flank HPTMs bound by NURF301 reader domains in peptide arrays, including H3K4me3, H3K9Ac, H3K9AcS10p, and H4K16Ac. NURF301 ChIP signals are well-correlated with H3K4me3, H3K9Ac, H3K9AcS10p, and H4K16Ac both at (**E**) TSSs and (**F**) genome-wide. (**G**) Heatmaps of NURF301, H3K4me3, H3K9Ac, H3K9AcS10p, and H4K16Ac signals relative to the TSS for genes ordered according to the NURF301 signal strength. NURF signals (purple) flank histone modification signals (green) at TSSs.

Taken together, these data suggest that the BPTF/NURF301 subunit of NURF can utilize two domains to interact with at least seven HPTMs. To confirm if these HPTMs are bound by NURF *in vivo*, we used ChIP-sequencing to profile the distribution of NURF in *Drosophila* S2 cells relative to a subset of these modifications: H3K4me3, H3K9Ac, H3K9AcS10p, and H4K16Ac. S2 cells were used as they are a hemocyte-derived cell line [41] from which large numbers of cells can be obtained, a key determinant of successful remodeler ChIP, while also allowing cross-comparison with genomics datasets from primary hemocytes. NURF ChIP-seq peaks were ranked into quintiles according to strength of associated ChIP signal, and HPTM distribution profiled relative to these NURF peaks. As shown in Fig. 3A, NURF associated with the selected HPTMs, and the strength of NURF ChIP signal was positively correlated with levels of HPTMs to which NURF binds. As an additional test of association between NURF and HPTMs, we isolated mononucleosomes from *Drosophila* S2 cells, immunoprecipitated NURF and associated nucleosomes, and analysed HPTM enrichment in NURF-associated nucleosomes by western blotting. Following normalization of input and bound fractions to total histone H3 levels, clear enrichment in the H3K4me3, H3K9Ac, H3K9AcS10p, and H4K16Ac combinatorial marks in the NURF-bound fraction was observed (Fig. 3B), confirming that NURF associates *in vivo* with nucleosomes containing these HPTMs. In contrast, NURF-bound nucleosomes lacked the inhibitory switch modification H3T3p.

H3K4me3 and histone acetylation are marks of active transcription, and consistent with this, we observed by ChIP-seq that H3K4me3, H3K9Ac, H3K9AcS10p, and H4K16Ac peak immediately downstream of the TSS at the +1 nucleosome (Fig. 3C,D, and E). Decoration of the +1 nucleosome with combinations of HPTMs that can be bound by NURF301 in turn resulted in the recruitment of NURF. We observed NURF301 ChIP-seq signals adjacent to HPTM peaks, immediately upstream of the +1 nucleosome (Fig. 3C and D). Good correlation was observed between NURF301 and HPTMs bound by NURF both at TSSs (Fig. 3E) and genome-wide (Fig. 3F). Furthermore, heatmap profiling of NURF and HPTM ChIP signals confirmed that NURF makes crosslinkable DNA contacts adjacent to the +1 nucleosome (Fig. 3G), consistent with NURF301 binding decorated histone tails on the +1 nucleosome via the C-terminal PHD2 finger and bromodomain, and with DNA contacts being made with the flanking linker DNA through N-terminal AT-hook region that is required for full nucleosome sliding activity [14].

### Live imaging reveals histone modifications are required for NURF-chromatin binding

**Figure 4.**
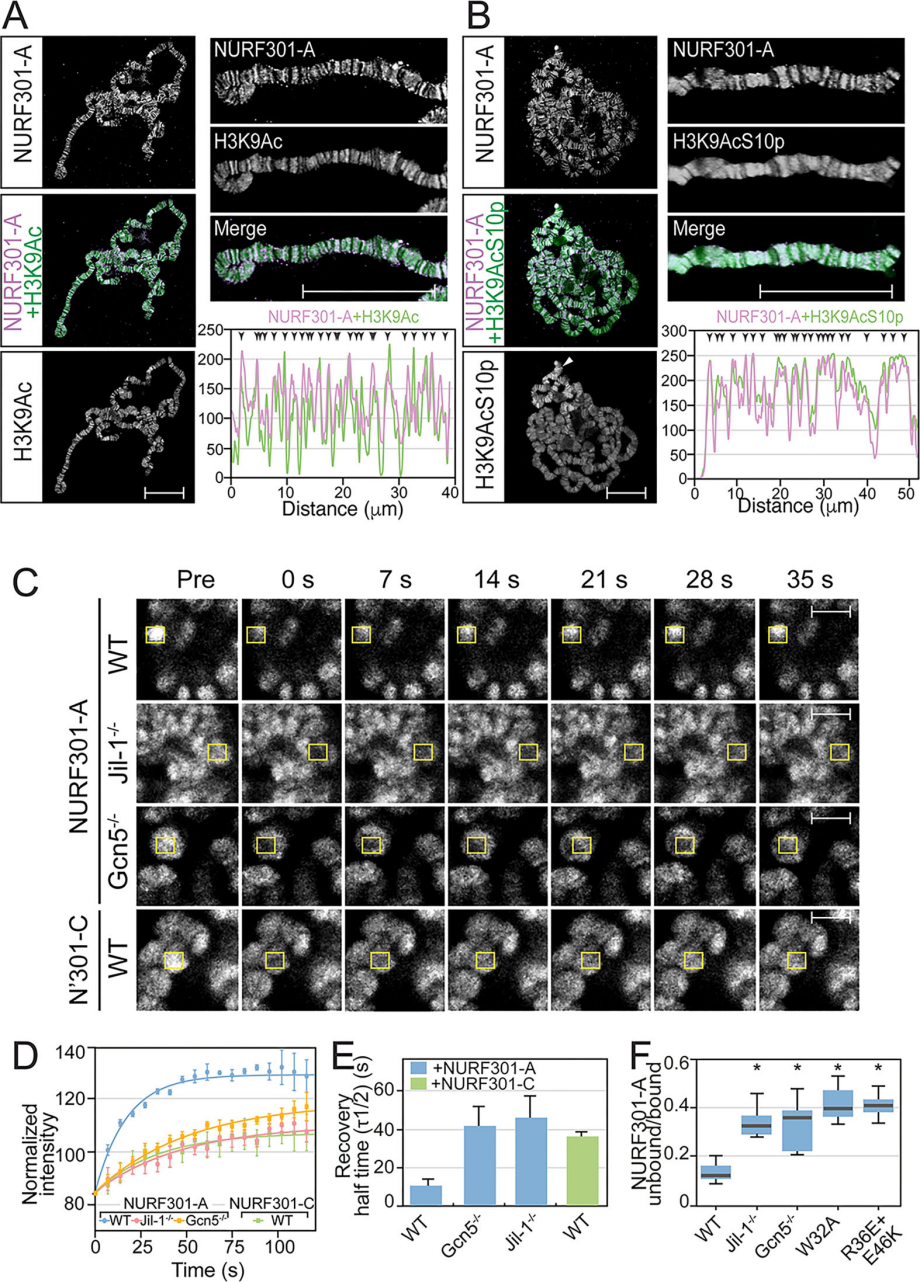
Polytene chromosome analysis shows NURF recruitment requires histone modification recognition for tight binding. Double labelling of salivary gland polytene chromosomes shows that GS-TAP-tagged full-length NURF301 (NURF301-A isoform; revealed by anti-protein G staining) colocalizes with histone (**A**) H3K9Ac and (**B**) H3K9AcS10p modifications. In merged images, NURF301-A is shown in purple and HPTMs in green, with overlap revealed in white. White arrowheads in panel (B) indicate the male X chromosome. Higher-magnification images of the chromosome arms are shown on the right to better discriminate colocalizing bands. Graphs show line scan of NURF301 (purple) and HPTM (green) signals across the chromosome arms in high-magnification images. Black arrowheads indicate regions where NURF301 and HPTM signals overlap. Scalebar in panels (A) and (B) represents 25 μm. (**C**) Real-time, post-bleach recovery of YFP-tagged NURF301 isoforms in salivary gland polytene chromosomes. Strains expressing YFP-tagged full-length NURF301-A isoform and the truncated NURF301-C isoform, which lacks the HPTM-binding reader domains, were used. YFP-NURF301-A FRAP was performed in *w^1118^* (WT) and *Jil-1* and *Gcn5* mutant backgrounds that lack H3S10p and H3K9Ac, respectively. Bleach regions are indicated by a yellow box. (**D**) FRAP recovery curves showing NURF301 recovery for 120 s after bleach. Scalebar represents 10 μm. (**E**) Bar-graph showing calculated half-time of NURF301 recovery. Data are mean and standard deviation of at least five determinations. (**F**) Live imaging of salivary glands containing YFP-tagged NURF301-A reveals that histone modification recognition is required for chromatin targeting. YFP-tagged NURF301-A transgenic larvae were imaged in *w^1118^* and *Jil-1* and *Gcn5* mutant backgrounds, as well as YFP-tagged NURF301-A larvae that contain mutations in the H3K4me3 recognition pocket (W32A) and the H3K9AcS10p-binding surface (R36E and E46K). By calculating the ratio of signal intensities of NURF301-A bound to chromatin (polytene bands) relative to staining off-chromatin in the nucleoplasm, efficiency of chromatin targeting could be determined. Data are mean and standard deviation of at least 100 determinations.

NURF–HPTM interactions were also supported by polytene chromosome analysis, where extensive overlap between NURF and the H3K9Ac and H3K9AcS10p marks was detected on all chromosome arms (Fig. 4A and B). To determine whether marks were required for NURF recruitment, we generated YFP-tagged WT full-length NURF301 as well as variants in which the PHD2 domain was mutated for H3K4me3 recognition (W32D) or contained double mutations in the H3K9AcS10p recognition surface (R36E and E46K). WT NURF301-YFP was also crossed into genetic backgrounds lacking H3K9Ac (*Gcn5* deletion strain [22]) or H3S10p (*Jil1* deletion strain [42]). Live imaging of salivary gland nuclei showed that WT NURF301-YFP binds discrete loci on chromosomes with limited staining of the nucleoplasm. In contrast, deletion of Jil-1 or Gcn5, or mutation of PHD2 K4me3 or H3K9AcS10p recognition, delocalized NURF. Bands could still be detected on polytene chromosomes, but elevated staining in the nucleoplasm was observed (Fig. 4F and Supplementary Fig. S3).

To verify whether binding of HPTMs was required for NURF recruitment to sites on chromatin, we performed FRAP experiments on polytene salivary glands using full-length NURF301-YFP strains as well as C-terminally truncated NURF301-YFP that lack all reader domains. Experiments were performed in WT as well as *Gcn5* and *Jil1* deletion strains that lack the H3K9Ac and H3S10p modifications, respectively (Fig. 4C–E). The use of polytene chromosomes allowed recovery kinetics of chromatin-bound NURF to be discriminated, as individual polytene chromosomal bands can be sampled/bleached. Full-length NURF301-YFP exhibited rapid recovery after photobleaching, with a half-life of recovery of 10.7 s (Fig. 4C–E). Removal of H3K9Ac and H3S10p in *Gcn5* and *Jil1* deletion strains, or removal of the NURF301 C-terminal reader domains (NURF-C in Fig. 4), delayed rebinding of NURF to loci (Fig. 4C–E) and increased half-life of recovery of at least four-fold (Fig. 4E).

**Figure 5.**
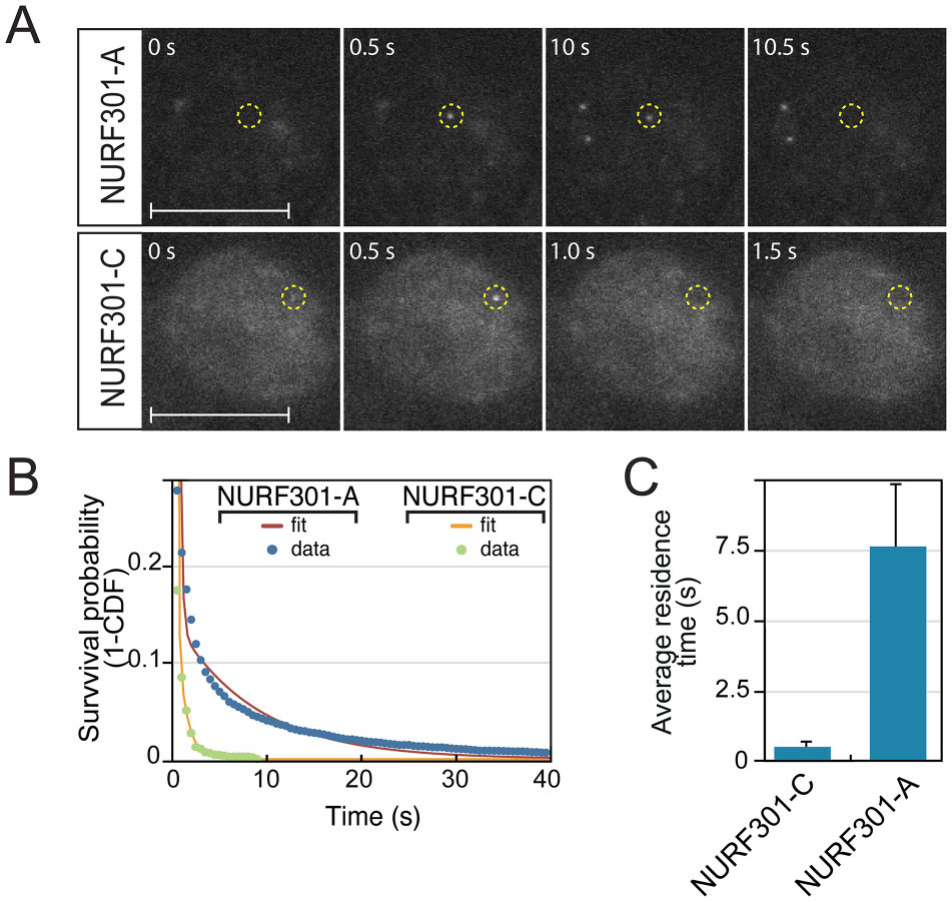
Single molecule imaging reveals stable NURF chromatin binding requires histone modification-binding domains. (**A**) HaloTag fusions with the full-length NURF301-A isoform or the NURF301-C isoform, which lacks the HPTM-binding reader domains, were expressed in cells, labelled with 0.5 nM JF646 HaloTag ligand, and nuclei imaged at 500 ms intervals to motion-blur unbound particles and resolve stably bound NURF complexes. Example spots for each NURF301 variant are displayed with frames before, during, and after single binding events. Scalebar represents 5 μm. (**B**) Plot showing uncorrected survival probability of single NURF301-A and NURF301-C HaloTag fusion molecules with two-exponential fits. (**C**) Bar chart showing average residence times of NURF301-C- and NURF301-A-HaloTag fusions, confirms histone modification recognition enhances residence time on chromatin.

FRAP recovery curves represent the behaviour of a large population of molecules. To provide an independent assessment of chromatin binding of NURF and the influence of HPTMs, we analysed binding kinetics of individual NURF complexes using NURF301-HaloTag fusion constructs and single-particle tracking. Hemocytes expressing HaloTag fusions with full-length (NURF301-A) and C-terminally truncated (NURF301-C) NURF301 isoforms under the control of the native promoter were labelled using JF-646 HaloTag ligand [43], and individual nuclei imaged to determine residence times of NURF complexes that can or cannot bind to HPTMs. Representitive nuclei are shown in Fig. 5A, where individual spots corresponding to bound NURF301-A and NURF301-C isoforms are presented. NURF301-A isoforms exhibit comparatively long residence times, in the example up to 9.5 s, while for NURF301-C isoforms, spots are seldom present for more than a single (500 ms) frame, and background corresponding to motion-blurred labelled NURF complexes was higher. By analysing at least 10 000 tracks for each construct, we could determine average residence time on chromatin. Residence times for NURF remodeler complexes that can bind HPTMs were determined to be ~7.5 s (Fig. 5C and Supplementary Movie 1). By contrast, NURF complexes that lack reader domains were highly dynamic with mean residence time <500 ms (Fig. 5C and Supplementary Movie 2).

### NURF remodeling targeted to nucleosomes enriched with NURF-bound histone modifications

Our imaging data were largely consistent with HPTMs enabling stable recruitment of NURF to genome targets. However, it was unclear whether recruitment due to HPTM-binding, in turn, facilitated enhanced nucleosome sliding and remodeling activity. To explore this question, we turned to the TSS-adjacent, downstream nucleosomes on active genes, which we showed earlier (Fig. 3C and G) are decorated with HPTMs bound by NURF. Previous studies have suggested that the identity of general transcription factors associated with core promoters of active genes can programme distinct profiles of transcription initiation, but also levels of HPTMs on downstream nucleosomes on active genes. In particular, housekeeping promoters have high levels of H3K4me3 and can be identified by the presence of DRE, Ohler box 1, and Ohler box 7 motifs and have dispersed patterns of initiation. Conversely, developmental/regulated promoters have low H3K4me3 levels and can be distinguished by the presence of TATA and Inr motifs and have focused initiation [44, 45]. As these two categories of active promoter show distinct H3K4me3 distributions, comparisons between them allow the effects of HPTMs on NURF recruitment and activity to be investigated and disentangled from the effects of transcription *per se*.

**Figure 6.**
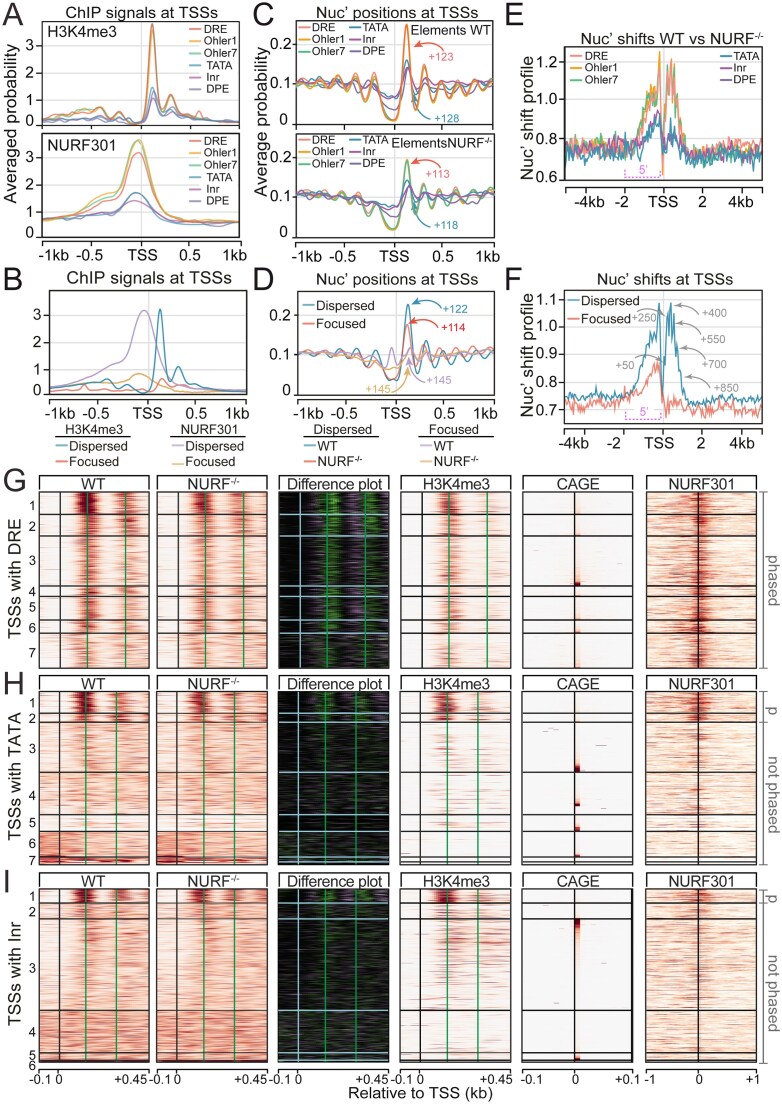
Enhanced NURF recruitment and activity at promoters enriched for histone modifications. Active promoters in hemocytes identified by CAGE were classified according to (**A**) promoter sequence elements or (**B**) initiation profile (dispersed versus focused), and associated H3K4me3 and NURF301 ChIP signals were analysed. Averaged nucleosome profiles surrounding promoters classified by (**C**) promoter elements or (**D**) initiation pattern. Promoters with high H3K4me3 and NURF301 show well-positioned nucleosomes downstream of the TSS. Averaged profiles of NURF-dependent nucleosome shifts at promoters classified by (**E**) promoter elements or (**F**) initiation pattern. (**G**–**I**) Heatmap analysis of nucleosome positioning, histone modifications, and NURF301 ChIP signals downstream of TSSs across promoter subclasses. Nucleosome probability was plotted from −100 to +450 bp relative to CAGE-defined TSSs in WT and *Nurf301* mutant (NURF^−/−^) hemocytes. Difference plots highlight nucleosome position changes between mutants (purple) or WT (green). TSSs were k-means clustered based on WT nucleosome signals and grouped by promoter motifs: (G) DRE, (H) TATA, and (I) Inr. Black vertical lines denote TSSs and green vertical lines indicate nucleosome positions.

We used *Drosophila* third-instar larval hemocytes as a convenient primary cell system to perform this analysis and first conducted CAGE sequencing [46] on WT and *Nurf301* mutant larval hemocytes precisely to define active TSSs. TSSs were then further classified according to flanking core promoter motifs [47] as well as the pattern of initiation (dispersed versus focused as described in [48]) to segregate TSSs into housekeeping versus developmental/regulated promoters. We then profiled HPTM and NURF301 distribution as well as nucleosome positions in WT and *Nurf301* mutant cells to determine contributions of HPTMs to NURF recruitment and chromatin remodeling activity. Consistent with previous data, we observed high levels of H3K4me3 on active housekeeping promoters in hemocytes (Fig. 6A, DRE, Ohler box 1, and Ohler box 7 motifs; Fig. 6B, dispersed initiation). In contrast, active developmental/regulated promoters showed significantly lower levels of H3K4me3 (Fig. 6A, TATA and Inr motifs; Fig. 6B, focused initiation). Higher levels of H3K4me3 on housekeeping promoters were accompanied by elevated H3K9Ac, H3K9AcS10p, and H4K16Ac, with levels conversely reduced on developmental/regulated promoters (Supplementary Fig. S4A, compare dispersed with focused promoters; Supplementary Fig. S4B, compare DRE/Ohler 1/Ohler 7 with TATA/Inr). In turn, increased deposition of H3K4me3, H4K16Ac, and the H3K9AcS10p rheostat modifications on housekeeping promoters was correlated with higher NURF301 signal (Fig. 6A, compare DRE/Ohler 1/Ohler 7 with TATA/Inr, Fig. 6B compare dispersed with focused promoters), consistent with these HPTMs directing NURF recruitment.

Differences in HPTMs and NURF recruitment on these distinct promoter types was associated with differential NURF nucleosome remodeling activity. Comparison of whole-genome nucleosome positions in WT and *Nurf301* mutants, determined by MNase-seq [11], revealed that promoters with the highest levels of NURF301 and NURF-bound HPTMs exhibited regular arrays of up to seven well-positioned nucleosomes downstream of TSSs in WT hemocytes [Fig. 6C (DRE and Ohler box), Fig. 6D (dispersed), Supplementary Fig. S4C (compare DRE and TATA)]. This was most obviously reflected in heatmaps of nucleosome position flanking TSSs, where clearly positioned nucleosome arrays could be observed downstream of the majority of DRE-containing TSSs in WT (Fig. 6G and Supplementary Fig. S4E). Heatmaps of H3K4me3 and NURF301 ChIP signal and CAGE tag counts flanking these TSSs indicated that nucleosome phasing was correlated with high levels of H3K4me3 and NURF301, but not necessarily the level of transcription (Fig. 6G and Supplementary Fig. S4E). In contrast, most developmental/regulated TSSs, which have low H3K4me3, did not exhibit well-positioned nucleosomes (Fig. 6H and I). A small number of promoters categorized as developmental/regulated exhibited well-positioned nucleosomes, but these also displayed high levels of H3K4me3 and NURF301 [Fig. 6H (k cluster 1 + 2), Fig. 6I (k cluster 1)].

Next, we discriminated sites of NURF remodeling/nucleosome sliding activity by determining regions where nucleosome position changed between WT and *Nurf301* mutants. This analysis confirmed that nucleosomes enriched in HPTMs were sites of NURF activity, establishing the link between HPTM levels, NURF recruitment, and nucleosome remodeling activity. Thus, on active promoters with high levels of HPTMs, the position of the +1 nucleosome shifted towards the TSS in *Nurf301* mutants, with shifts propagated and increased over the subsequent six nucleosomes (Fig. 6C and D; Supplementary Fig. S4C). This was confirmed by comparing the averaged profiles of NURF shifts flanking TSSs of different promoter classes (Fig. 6E and F). NURF-dependent nucleosome shifts were calculated by measuring normalized tag densities of uniquely mapped nucleosome reads in a sliding 50 bp window across the genomes of WT and *Nurf301* mutant hemocytes and comparing log_2_ fold changes between WT and *Nurf301* mutants. Promoters with highest levels of H3K4me3 and NURF (DRE, Ohler box 1, Ohler box 7, or dispersed promoters) also exhibited most changes in nucleosome position downstream of the TSS between WT and *Nurf301* mutants, where repeated peaks in shifts corresponding to the linkers between the +1 to +6 nucleosomes downstream of the TSS were detected. The magnitude of these nucleosome shifts was greatest on genes that exhibited highest densities of NURF and NURF-bound HPTMs. All promoter classes, irrespective of H3K4me3, show changes in nucleosome position upstream of the TSS (5′ indicated), consistent with recruitment of NURF to enhancers via TF-mediated interactions as observed previously.

As a further measure of NURF remodeling activity, we plotted heatmaps of the differences in nucleosome density between WT and *Nurf301* mutant cells downstream of TSSs of each promoter class. This comparative difference heatmap of nucleosome positions in WT and *Nurf301* mutants revealed clear directional movement, on promoters with the highest levels of HPTMs and NURF301 ChIP signal, of nucleosomes towards the TSS in *Nurf301* mutants (Fig. 6G–I, Difference plot), suggesting that NURF normally acts on these promoters to move nucleosomes away from the TSS. Thus, TSSs that contain DRE elements (Fig. 6G) exhibited well-positioned nucleosomes downstream of the majority of TSSs of this class with nucleosome difference plot clearly indicating nucleosome shifts towards the TSS in *Nurf301* mutants. All nucleosomes showed high +1 nucleosome H3K4me3 ChIP signal and flanking NURF301 ChIP signal. In contrast, the majority of TATA-containing TSSs (Fig. 6H) did not exhibit H3K4me3 and NURF301 ChIP-signal or well-positioned nucleosomes and showed few changes in nucleosome organization in *Nurf301* mutants. A subset of promoters (k-clusters 1 + 2) exhibited well-positioned nucleosomes, but this correlated with higher levels of H3K4me3 and NURF301 ChIP signal and corresponding shifts in nucleosome positions in *Nurf301* mutants. Likewise, Inr-containing TSS (Fig. 6I), with the exception of k-cluster 1, did not show significant H3K4me3 and NURF301 enrichment, well-positioned nucleosomes, or significant changes in nucleosome organization in *Nurf301* mutants.

Taken together, these results suggest that a combination of HPTMs together discriminate the +1 nucleosome on subsets of active genes and mediate recruitment of the NURF remodeling enzyme. NURF then acts to maintain position of the +1 nucleosome as well as maintain correct spacing of at least six nucleosomes downstream of the TSS on these subsets of active genes.

### NURF does not influence transcription initiation site selection but regulates transcriptional output

In principle, these movements of the +1 nucleosome towards the TSS in *Nurf301* mutants, and changes in spacing between subsequent downstream nucleosomes, have the potential to impact both the normal transcription initiation site but also lead to initiation at ectopic sites in the coding regions of genes—so-called cryptic initiation. We therefore tested whether correct positioning of nucleosomes downstream of the TSS by NURF was required to regulate transcription and, if so, at what stage of the transcription cycle this activity was needed. We compared CAGE data from WT and *Nurf301* mutant *Drosophila* larval hemocytes, quantifying changes both in the location of transcription initiation and the level of transcription.

**Figure 7.**
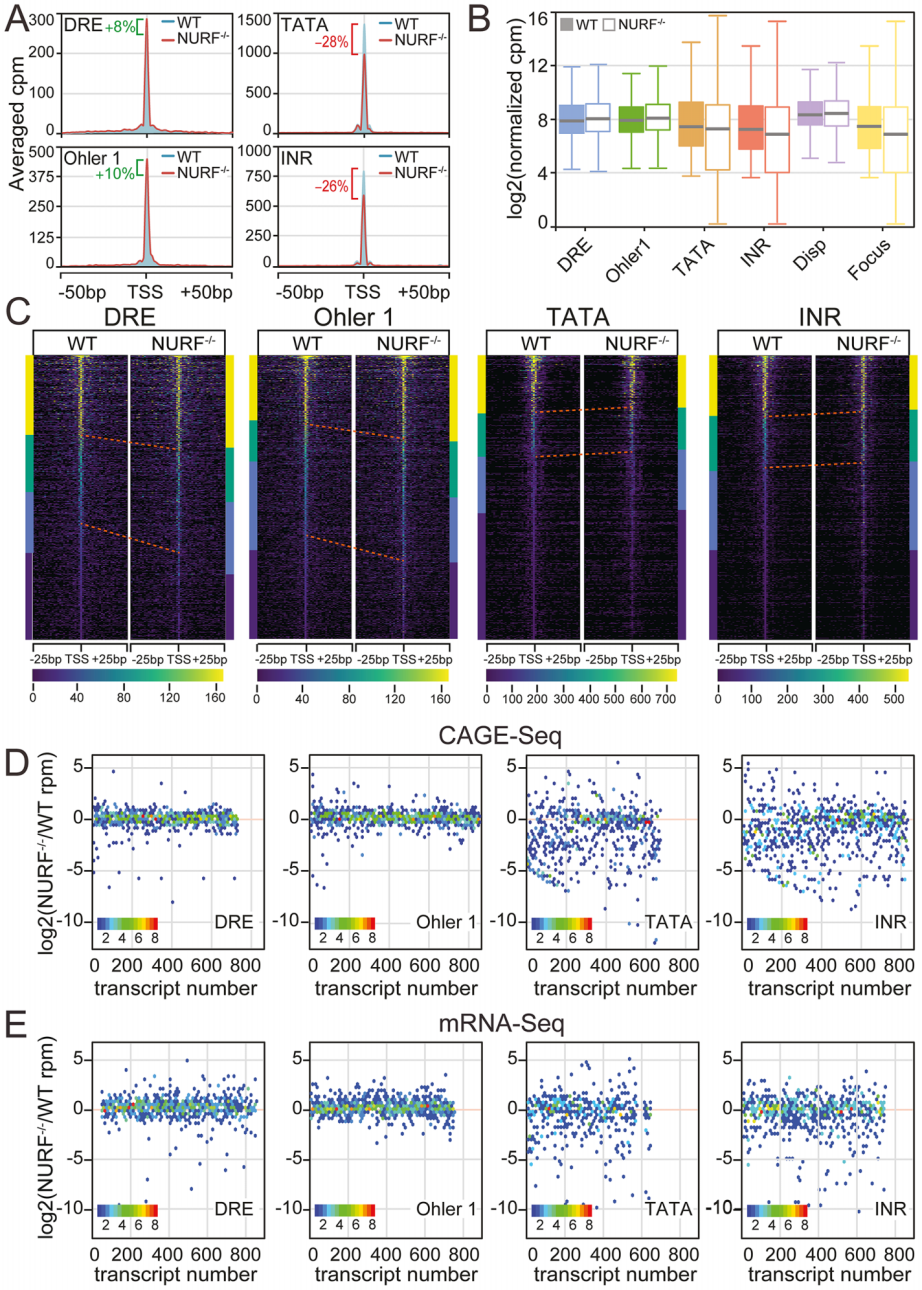
NURF does not influence transcription initiation site but does regulate transcript output. (**A**) Averaged profiling of CAGE tags from WT and *Nurf301* mutant (NURF^−/−^) hemocytes relative to main transcription initiation site, defined from WT CAGE data for TSSs of housekeeping genes (DRE, Ohler 1) and developmental/regulated promoters (TATA, Inr). (**B**) Boxplot showing normalized CAGE tag counts at TSSs of promoter classes. *Nurf301* mutant CAGE read numbers were normalized to WT CAGE. (**C**) Heatmap of normalized CAGE reads upstream and downstream of TSSs reveals no change in initiation site in *Nurf301* mutants but confirms increased expression at housekeeping TSSs and decreased expression of developmental/regulated TSSs. (**D**) Hexbin map of log_2_ fold change in normalized CAGE reads between WT and *Nurf301* mutants at different TSS subclasses. (**E**) Hexbin map of log_2_ fold change in normalized mRNA-seq reads between WT and *Nurf301* mutants at different TSS subclasses. In all hexbin maps, transcripts in either WT or *Nurf301* mutants were categorized according to TSS elements—either DRE, Ohler 1, TATA, or Inr elements—ordered according to genomic location and assigned a numerical ID—in the case of CAGE-seq with DRE from 1 to 738. Plots show log_2_ fold change in normalized expression versus transcript number, and plot tiles are coloured according to number of promoters/genes with similar expression changes as indicated in accompanying scalebars. In all hexbin maps orange line indicates no change in expression.

Contrary to expectation, we did not detect any changes in the location of the normal TSS in *Nurf301* mutants on any promoter class by either averaged (Fig. 7A) or heatmap profiling (Fig. 7C) of CAGE signals. Moreover, we did not detect any evidence of cryptic initiation from ectopic sites in gene bodies in *Nurf301* mutants. However, although TSS location remained the same in *Nurf301* mutants, transcriptional output was altered. These changes segregated into two distinct classes that reflected our previous categorization of housekeeping (high-H3K4me3/high-NURF/dispersed-initiation) and developmental/regulated promoters (low-H3K4me3/low-NURF/focused-initiation). Surprisingly, housekeeping promoters, which contain the highest levels of promoter-proximal H3K4me3, NURF301, and NURF-dependent nucleosome sliding, showed slightly elevated transcription levels both by averaged profile plot (Fig. 7A, DRE-8% increase, Ohler 1–10% increase; Supplementary Fig. S4G), boxplot (Fig. 7B), heatmap (Fig. 7C), and hexbin map (Fig. 7D, majority of CAGE tags lie above zero change threshold). In contrast, developmental/regulated promoters that lacked high promoter-proximal NURF activity showed dramatic reduction in transcription, as revealed by averaged profile plot (Fig. 7A, TATA-28% reduction, Inr-26% reduction; Supplementary Fig. S4H), boxplot (Fig. 7B, decreased median expression), heatmap (Fig. 7C, reduced expression across expression categories), and hexbin map (Fig. 7D, majority of CAGE tags lie below zero change threshold). These trends were confirmed by mRNA-seq of WT and *Nurf301* mutants, where results for the different promoter classes were indistinguishable from that for CAGE-seq data (compare Fig. 7D and E).

These data are broadly consistent with previous analyses showing that transcription in *Drosophila* does not directly depend on histone modification [49, 50] and suggest, instead, a more subtle relationship between promoter-proximal HPTMs, NURF nucleosome sliding, and transcription. Some indications of this could be found by examining the impact of paused RNA polymerase II (Pol II) stability on chromatin organization and NURF dependencies. We used an alternative approach to classify our CAGE-determined, hemocyte-expressed TSSs according to previously described metrics of paused Pol II stability [51], segmenting active TSSs into five quintiles ranging from those where Pol II release occurs rapidly (Quintile 1) to those where Pol II is stably paused (Quintile 5). By comparing stable-pause promoters with labile promoters, we can, in principle, distinguish the impact of transcription initiation from the downstream events of Pol II elongation and +1 nucleosome transit. This analysis revealed that detectable promoter-proximal H3K4me3 or NURF301 levels were inversely correlated with paused Pol II stability. Thus, TSSs with labile Pol II displayed higher levels of both H3K4me3 and NURF301 (Supplementary Fig. S5A), as well as with higher NURF-dependent nucleosome remodeling (Supplementary Fig. S5B). In contrast, remodeling was largely absent downstream of the TSS on promoters with the most stable paused Pol II (Supplementary Fig. S5B). Moreover, well-positioned promoter-proximal nucleosomes (+1 to +5 nucleosomes) could be detected at TSSs with labile Pol II, while TSSs with the most stably paused Pol II displayed poorly phased promoter-proximal nucleosomes with the exception of the +1 nucleosome (Supplementary Fig. S5D and E).

These differences were associated with altered transcript output both in WT hemocytes as well as differences in dependence on functional NURF complex. Thus, as noted previously [51], TSSs with labile paused Pol II exhibited higher mean steady-state expression in WT compared to TSSs with stably paused Pol II (Supplementary Fig. S5C, compare WT quintile 1 and 5). In *Nurf301* mutants, expression from TSSs with labile paused Pol II expression was increased relative to WT (Supplementary Fig. S5C, quintiles 1–4), but reduced at TSSs with stably paused Pol II (Supplementary Fig. S5C, quintile 5). Cumulatively, these data emphasise promoter-proximal functions for both HPTMs and NURF remodeling, not in transcription initiation but rather in escape and/or dissociation of paused polymerase. These are separable from remodeler activities on 5′ regulatory regions of genes where NURF facilitates actions of sequence-specific TFs in transcription initiation.

## Discussion

By controlling nucleosome dynamics, ATP-dependent chromatin remodeling enzymes like NURF have the potential to regulate multiple chromatin-templated transactions ranging from transcription to repair and replication. A key component to discriminating the discrete functions of specific remodeling complexes is an improved understanding of the nature of their individual nucleosomal substrates and how nucleosome diversity can regulate both remodeler deployment to, and activity at, genomic loci. Here, we build upon previous work showing that the BPTF/NURF301 subunit of NURF recognizes the histone H3K4me3 and H4K16Ac marks [15–17], to demonstrate that NURF recognizes a combination of at least seven histone modifications on the histone H3 and H4 tails. We show this is mediated by a ‘reader-head’ on BPTF/NURF301 that comprises the PHD2 domain, which binds triply modified H3 tails bearing the H3K4me3K9AcS10p signature, and a bromodomain that recognizes tetra-acetylated histone H4 tails. We demonstrate, using genetics, genomics, and single-molecule imaging methods that recognition of HPTMs by this module is required for stable recruitment of NURF to chromatin, which, in turn, is associated with NURF-dependent nucleosome sliding.

Our analysis expands the binding repertoire of the BPTF/NURF301 bromodomain that we and others previously showed binds H4K16Ac [16, 17], by demonstrating enhanced binding to tetra-acetylated H4 compared with the single H4K16Ac mark. These data are entirely consistent with previous characterization of BRD4 family bromodomains where high-affinity binding requires multiply acetylated histone tails [52, 53]. While crystal structures of these interactions indicate that BRD4 family bromodomains can only bind to two acetylated residues at any one time, binding to multiply acetylated tails is likely stabilized by rapid exchange between flanking di-acetylated residues [52, 54]. Our measured *in vitro* affinity of the BPTF/NURF301 bromodomain for tetra-acetylated H4 (*K*_d_ = 96 μM) is consistent with previous measurements of affinities of single bromodomains for modified histones, but places the bromodomain at the lower end of the spectrum of binding affinities for histone reader domains [55]. This weak interaction implies that H4 tail recognition by the BPTF/NURF301 bromodomain, even when stimulated by multiple acetylation, is unlikely by itself to be a major driver of *in vivo* recruitment.

In contrast, we show that the BPTF/NURF301 C-terminal PHD finger (designated PHD2 in these studies) binds with high affinity (300 nM) to the appropriately modified H3 tail substrates. An important element in this tight binding is the contribution of a novel binding pocket, identified by this study, that recognizes H3K9Ac and H3S10p modifications. This additional binding surface co-operates with the previously characterized, H3K4me3-binding, hydrophobic cage on the PHD2 domain [25] to increase binding strength from intermediate affinity (*K*_d_ = 2 μM), when single H3K4me3 modifications are present, to high affinity (*K*_d_ = 300 nM) for H3 tail peptides that contain the triple modification H3K4me3K9AcS10p. Given previous estimates of nuclear ISWI remodeler concentrations [56], affinities in this range are likely to result in high occupancy of NURF at binding targets. We expect this to be further enhanced by the simultaneous contribution of multivalent recognition of the tetra-acetylated H4 tail of the bromodomain to the avidity of interactions between NURF and modified nucleosomes through increases in valency [57, 58], in turn driving recruitment to substrate nucleosomes.

An obvious caveat is that our *in vitro* histone binding screens and studies were performed using isolated histone tail peptides and protein domains. However, confidence that the observed results reflect interactions that occur in the context of a nucleosome and/or the full NURF complex is provided by our analysis of native nucleosomes, chromatin immunoprecipitation profiles, and imaging of NURF on *Drosophila* chromosomes. In particular, we show that native nucleosomes bound by NURF are enriched in all the HPTMs we observed to enhance NURF binding, that NURF301 overlaps with the distribution of H3K4me3, H3K9Ac, H3S10p, and H4K16Ac by ChIP, and on polytene chromosomes. Furthermore, ablation of the enzymes responsible for deposition of the H3K9Ac and H3S10p marks decreases the binding of NURF301 to chromosomes *in vivo*. Cumulatively these data indicate that a signature of at least seven HPTMs directs stable binding of NURF to sites in chromatin *in vivo*. While by no means definitive, comparison by single particle tracking of residence times of NURF complexes that can bind HPTMs indicates a substantial increase in stability of chromatin interaction.

An important feature of the interactions we observe between both the PHD2 domain and bromodomain, and modified histone tails is that interactions are anchored by single modifications (H3K4me3 and H4K16Ac) that act as primary recognition sites, flanked by switch/rheostat modifications that modulate binding to the primary recognition site. This is clearly illustrated by the BPTF/NURF301 PHD2 domain where the primary recognition site is H3K4me3. We do not observe binding of the PHD2 domain to single H3K9Ac or H3S10p modifications in the absence of H3K4me3. However, H3K9AcS10p acts as a phospho-acetyl switch or rheostat that enforces a graded increase in binding, as has been suggested previously for other modifications and reader domains [59]. The enhancement of BPTF/NURF301 PHD2 binding by the H3K9AcS10p modification is entirely consistent with previous studies showing cross-talk between these modifications and their respective writers [60–65].

While the bulk of this report focuses on switch/rheostat modifications that increase binding of the PHD2 and bromodomain of NURF to chromatin, our peptide array screen also detected modifications that prevent histone tail recognition. In particular, we observed that H3T3p inhibited PHD2 recognition of H3K4me3, probably as a result of unfavourable charge interactions between H3T3p and negatively charged residues in the PHD2 H3K4me3 binding pocket. Negative switches like H3T3p provide a convenient mechanism by which binding of NURF to histone tails can be masked in a regulated manner, without requiring wholesale removal of positive modifications. Consistent with this, we have shown that NURF is ejected from chromatin during mitosis, which corresponds with the appearance of high levels of H3T3p and H3T3pK4me3 [39]. Likewise, our data show that H3R2me2a and H3R2Cit can block PHD2 recognition of H3K4me3, consistent with previous results [66, 67]. Understanding the dynamics of negative switches during cell and transcriptional cycles will be vital to elucidating their roles in regulating stage-dependent remodeler recruitment.

For positive-acting modifications, however, we propose that an ordered progression of histone modification, driven by cross-talk between H3K4me3, H3K9Ac, H3S10p, and H4K16Ac marks, and directed by stimulatory effects of H3K4me3 and H3S10p on Gcn5 [61, 62], H3K9AcS10p on MLL [60], and H3 phosphoacetylation on MOF [63–65], generates a multiply-modified nucleosome substrate for the NURF remodeler. Our data are most consistent with a tethering mechanism where interactions via two reader domains with two distinct histone tail signatures, H3K4me3K9AcS10p (PHD2) and H4K5AcK8AcK12AcK16Ac (bromodomain), in turn recruit NURF to promoter-proximal nucleosomes, where NURF positions the + 1 nucleosome and spaces downstream nucleosomes.

Surprisingly, this recruitment of NURF by HPTMs to remodel promoter-proximal nucleosomes has relatively modest consequences on bulk transcriptional output. In *Nurf301* mutants, promoters with high levels of active HPTMs, high NURF301, and significant NURF-dependent promoter-proximal remodeling activity showed no change in initiation site and only modest increases in transcription levels. Conversely, promoters with low levels of active HPTMs, low NURF301, and low NURF-dependent promoter-proximal remodeling showed the most significant changes in transcriptional output in *Nurf301* mutants. These data are broadly consistent with previous analyses showing that transcription does not directly depend on histone modification in *Drosophila* [49, 50] and highlight a more subtle relationship between promoter proximal HPTMs and transcription. Moreover, these results likely illuminate distinct roles for NURF remodeling at different stages of the transcriptional cycle on different classes of promoter.

Numerous studies have shown that promoters can be functionally segregated on the basis of core promoter motifs, patterns of initiation, and H3K4me3 levels into either housekeeping or developmental/regulated promoters [44, 45]. Our studies suggest that promoter-proximal NURF signals, NURF remodeling activity, and transcriptional outcome in *Nurf301* mutants (increased versus decreased expression) can be added to these criteria. Thus, housekeeping promoters typically have dispersed initiation, high H3K4me3, high promoter-proximal NURF recruitment and sliding, and increased transcription in *Nurf301* mutants; while developmental/regulated promoters have focused initiation, low H3K4me3, low promoter-proximal NURF recruitment and sliding, and decreased transcription in *Nurf301* mutants. These disparate transcriptional responses in *Nurf301* mutants most likely reflect action of NURF at distinct stages of the transcription cycle on housekeeping versus developmental/regulated promoters. Two modes of regulation are entirely consistent with our previous maps of NURF sliding activity in bulk hemocytes where we observed NURF activity both at promoter-proximal nucleosomes, as well as on 5′ regulatory elements, and flanking TF-binding sites [11]. We speculate that developmental/regulated promoters, which have complex regulatory elements, depend on NURF-remodeling activity to facilitate access of TF activators to upstream regulatory elements to initiate transcription. In contrast, we envisage that housekeeping promoters that typically lack complex upstream regulatory elements, rely on HPTMs and NURF to control transcription post-initiation to ensure transcriptional consistency across time and among cell populations (as has been suggested previously for H3K4me3 [68]). Certainly, the tight phasing of promoter-proximal nucleosome positions on housekeeping genes demonstrates tight consistency of nucleosome positions across all cells in the population that is lost in *Nurf301* mutants with accompanying changes in transcription. A major technical challenge going forward will be to determine the extent to which this phasing impacts transcription on individual genes across populations of cells.

Finally, while our data show positive correlation between HPTMs and the recruitment and function of NURF, previous reports have suggested an antagonistic relationship for at least some of these modifications. For example, H4K16Ac has been shown to inhibit ISWI ATPase activity [69] by preventing relief of the AutoN inhibitory domain of ISWI by the histone H4 tail [70]. Moreover, H3K4me3 can block interactions of the NURF-55 subunit of NURF with the histone H3 tail [21]. How can one resolve the apparent contradiction that BPTF/NURF301 binds to modified histone H3 and H4 tails, that NURF localizes with such modifications on chromatin, and that high levels of these HPTMs are associated with promoter-proximal NURF nucleosome remodeling, yet activity of NURF simultaneously requires unmodified H3 and H4 tails? One possible explanation for this conundrum is that the appropriate substrate for NURF may be an asymmetric nucleosome that contains both modified and unmodified tails. An asymmetric distribution of HPTMs would allow both binding and simultaneous stimulation of activity. Indeed there is evidence of asymmetry in the distribution of histone marks [71] and underlying DNA contacts [72] especially at the +1 nucleosome in yeast. This is supported by studies showing interaction of writer enzymes with asymmetrically modified nucleosome substrates [73, 74].

Nucleosome asymmetry also provides a convenient mechanism by which directionality of sliding by ATP-dependent chromatin remodelers like NURF could be achieved. In the absence of other orienting cues (for example variable DNA linker lengths) remodelers can engage both faces of symmetric nucleosomes, alternatively sliding nucleosomes in opposite directions with no net translocation. However, asymmetric HPTMs would, in principle, allow remodeler deployment to one face of the nucleosome with resultant directional sliding. Our comparison of nucleosome positions in WT and *Nurf301* mutants certainly shows that NURF mediates directional sliding of nucleosomes at promoter-proximal nucleosomes. A key goal of future work will be to establish the extent to which such asymmetry exists and contributes to remodeler specificity.

## Supplementary Material

gkag494_Supplemental_Files

## Data Availability

Sequence data are available as SRA submissions (PRJNA737639, PRJNA738309, and PRJNA738389).
