# Supplementary material for: Combinatorial histone modifications direct ATP-dependent chromatin remodeling by NURF to promoter-proximal nucleosomes
Source: Nucleic Acids Res. 2026 May 21;54(10):gkag494. doi: 10.1093/nar/gkag494 (PMC13191287; doi:10.1093/nar/gkag494)
Supplement: gkag494_Supplemental_Files [file gkag494_supplemental_files.zip › Supplemental data and methods.docx]

**Supplementary Figures**

**
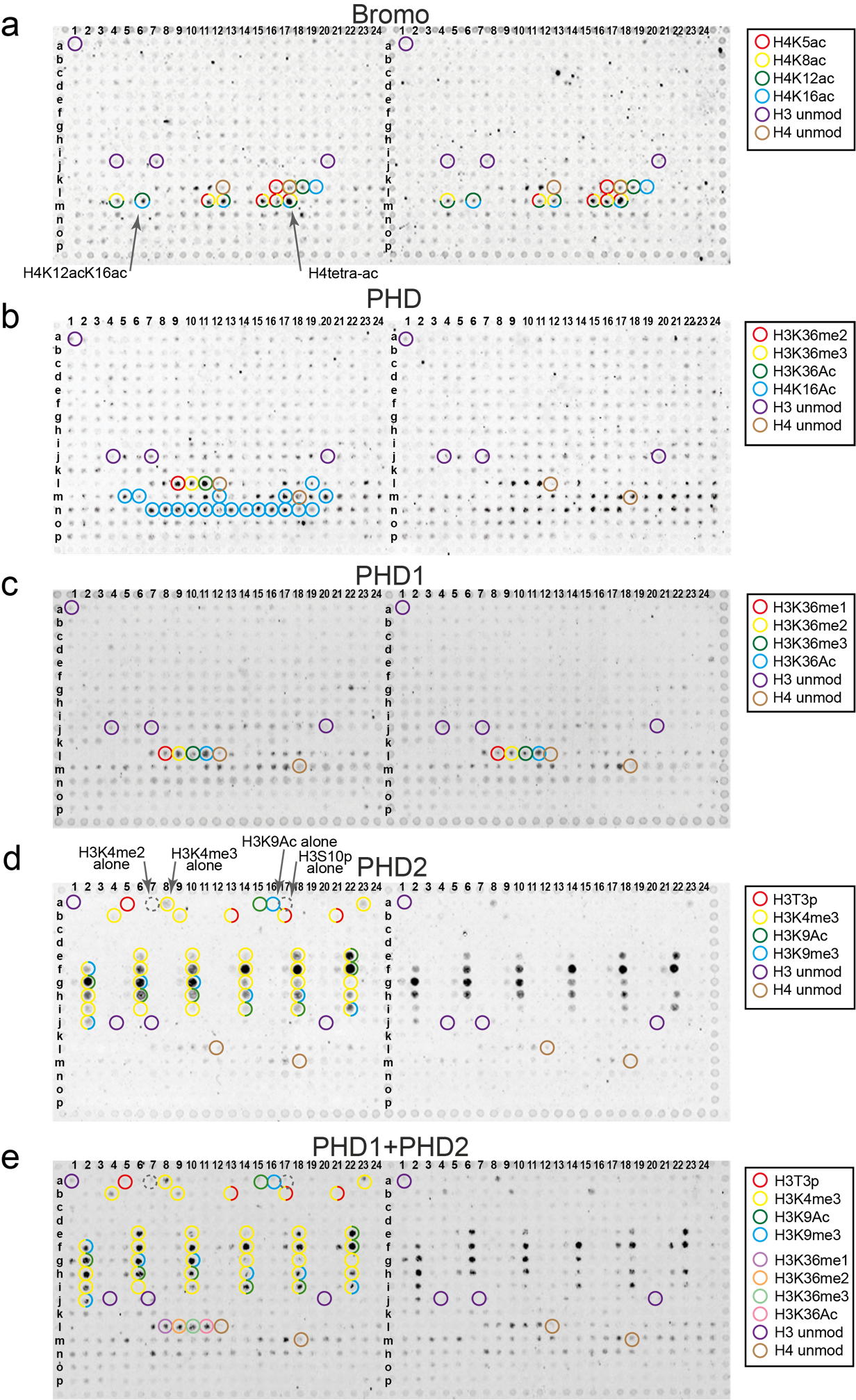
**

**Supplementary Fig. 1.** Binding of NURF301 reader domain-GST fusion proteins to modified histone peptide arrays. Modified histone peptide arrays containing different histone modification combinations were probed with (**A**) Bromodomain-GST fusion protein, (**B**) PHD-GST fusion protein, (**C**) PHD1-GST fusion protein, (**D**) PHD2-GST fusion protein, (**E**) the double PHD1-PHD2-GST fusion protein. Reader domain- GST fusion protein bound spots were detected using anti-GST HRP-conjugated primary antibody and IRDye 800CW-conjugated rabbit anti-HRP secondary antibody.

**
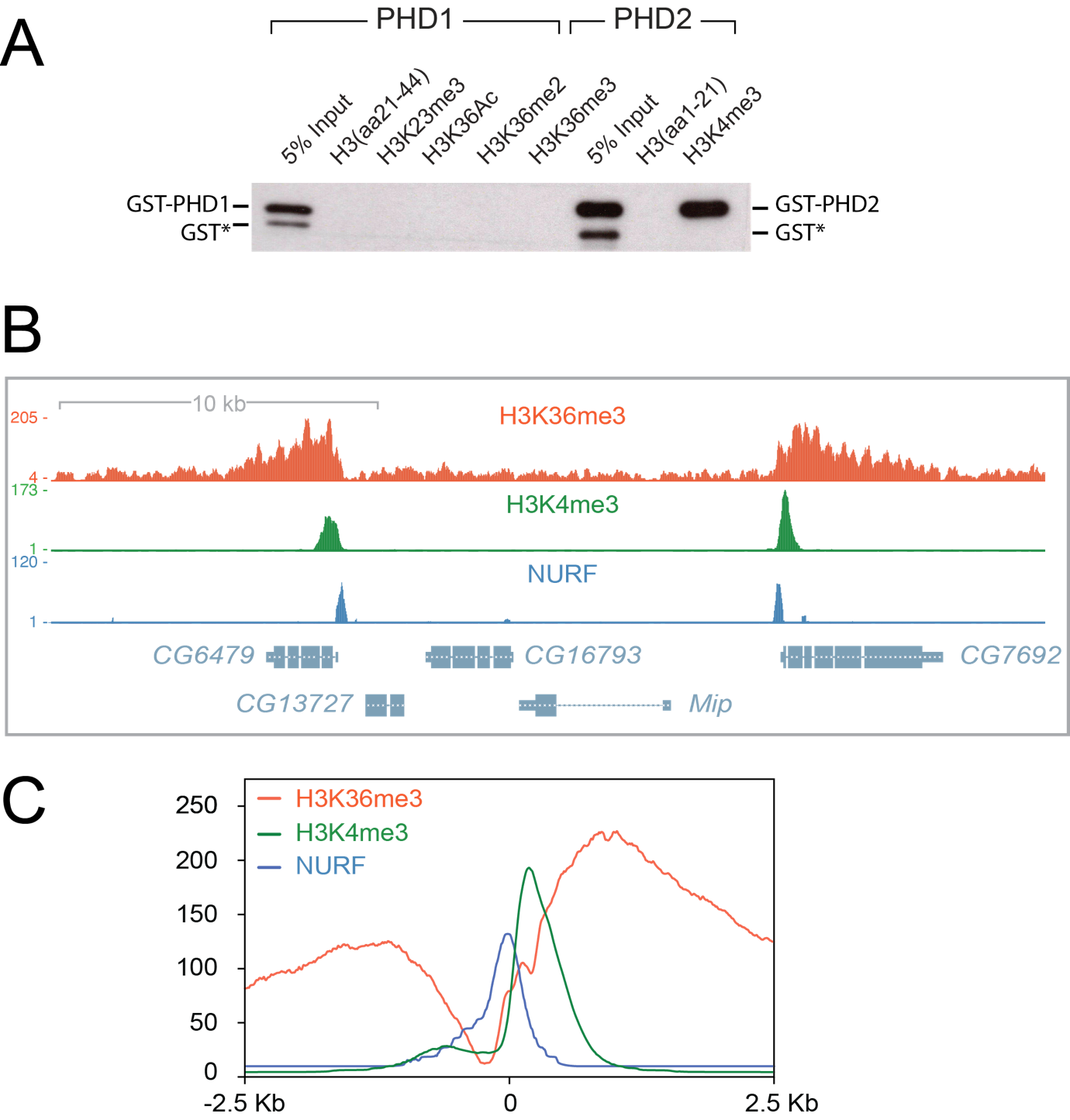
**

**Supplementary Fig. 2.** PHD1 domain peptide pull down assays. (**A**) *Drosophila* NURF301 PHD1-GST fusion protein failed to bind to histone modifications identified from modified histone peptide arrays. Peptide pull down and western blot analysis using HRP-conjugated anti-GST antibody was performed. Input lane shows full-length GST-PHD as well as a truncated product (GST*). As a control PHD2 binding to H3K4me3 is included. Comparison of NUR301 and H3Kme3 ChIP-seq data with publically available H3K36me3 data ({Jayakrishnan, 2025 #817}) show that while NUR301 flanks H3K4me3 it does not colocalise with the bulk of H3K36me3 on (**B**) individual genes or (**C**) at all TSSs

**Supplementary Fig. 3.** Live imaging of salivary glands containing YFP-tagged NURF301-A reveals that histone modification recognition is required for chromatin targeting. YFP-tagged NURF301-A transgenic larvae were imaged in wild-type (*w^1118^*) and *Jil-1* and *Gcn5* mutant backgrounds. In addition, YFP-tagged NURF301-A strains in which the PHD2 domain is mutated to disrupt the H3K4me3 recognition pocket (W32A) and the H3K9AcS10p binding surface (R36E and E46K) were imaged.

**
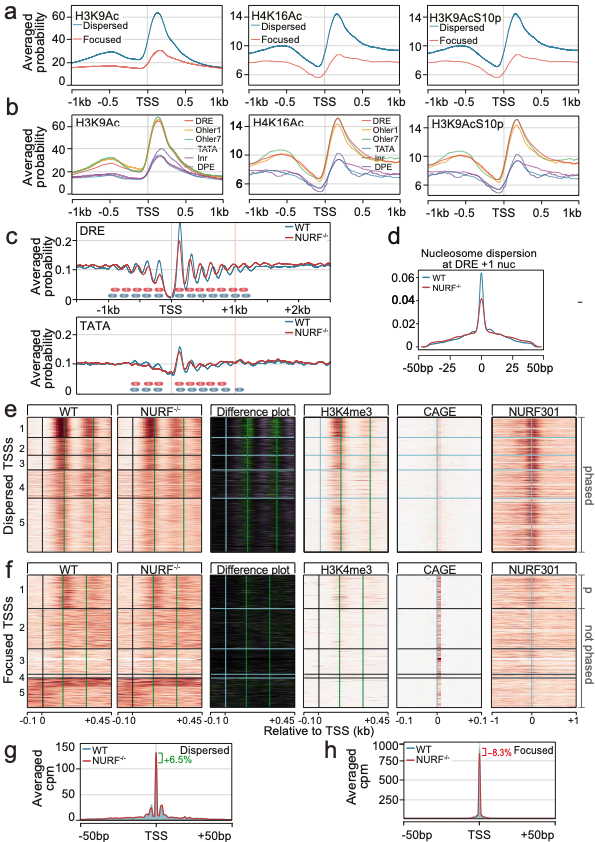
Supplementary Fig. 4.** Histone modification differences and transcriptional dependencies of TSS subclasses. **a,b** Active promoters in hemocytes were identified by CAGE and classified according to **a** profile of initiation (dispersed versus focused) or **b** sequence elements that characterise house-keeping (DRE, Ohler box 1, Ohler box 7) and developmental/regulated promoters (TATA, Inr, DPE), and flanking H3K9Ac, H4K16Ac and H3K9AcS10p ChIP signals profiled. **c** Averaged profiling of nucleosome positions flanking promoter sub-classes containing DRE or TATA elements. Nucleosome profiles were plotted relative to a point 1kb downstream of CAGE-determined TSSs (indicated by orange line), confirms that nucleosome phasing downstream of TSSs of active housekeeping genes is observed irrespective of the genomic viewpoint using for average profiling. Clear phasing of 7 nucleosomes downstream of the TSS is observed on DRE-containing promoters. Nucleosome position in both WT and *Nurf301* mutant hemocytes, are plotted. **d** Dispersion of +1 nucleosome position from the most abundant nucleosome position in the nucleosome ensemble DRE-containing promoters indicates that in addition to shifts towards the TSS, nucleosomes in *Nurf301* mutant hemocytes are dispersed from the peak location indicating “fuzzier” distribution. **e,f** Heatmap profiling of nucleosome positions, histone modifications and NURF ChIP signals downstream of TSSs with dispersed or focused patterns of initiation. Nucleosome probability in WT and *Nurf301* mutant hemocytes was plotted in a window from 100bp upstream to 450bp downstream of CAGE-determined TSSs of the indicated promoter classes. Changes in nucleosome position between WT and *Nurf301* mutant hemocytes were revealed by difference plot in which regions with higher nucleosome density in *Nurf301* mutant hemocytes were indicated in purple and regions with higher nucleosome density in WT hemocytes were shown in green. **e** TSSs that exhibit dispersed initiation typical of housekeeping genes showed well positioned nucleosomes downstream of the majority of TSSs of this class. Nucleosome difference plot clearly indicates nucleosome shifts towards the TSS in *Nurf301* mutant hemocytes. All nucleosomes showed high +1 nucleosome H3K4me3 ChIP signal and flanking NURF301 ChIP signal. Profiling of WT CAGE reads reveals that H3K4me3 and NURF301 levels does not correlate with absolute levels of transcription. **f** TSSs that exhibit focused initiation revealed that only a subset of such promoters exhibited well positioned nucleosomes. This subset of nucleosomes showed higher levels of +1 nucleosome H3K4me3 ChIP signal and NURF301 ChIP signal and corresponding shifts in nucleosomes towards the TSS in *Nurf301* mutant hemocytes. However, the majority of focused TSS failed to exhibit well positioned nucleosomes, H3K4me3 or NURF301 ChIP-signal and did not show significant changes in nucleosome organisation in *Nurf301* mutant hemocytes. **g,h** Averaged profiling of CAGE tags from wildtype (WT) and *Nurf301* mutant (NURF^-/-^) hemocytes relative to main transcription initiation site defined from WT CAGE data, reveals loss of NURF does not affect transcription initiation site but does affect transcriptional output, with elevated levels of transcription from TSSs with **g** dispersed initiation and reduced transcription from TSSs with **h** focused initiation.

**
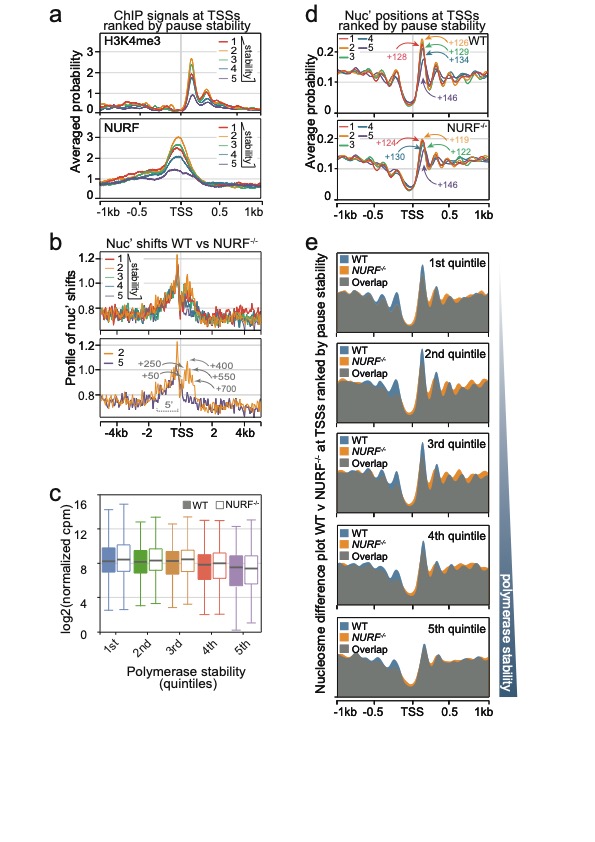
**

**Supplementary Fig. 5.** Histone modification differences and transcriptional dependencies of at promoters with paused polymerases ranked by pause stability. Active promoters in hemocytes were identified by CAGE and classified according to stability of paused RNA polymerase II based in previously published datasets^1^ and divided into 5 quintiles from least stable (1) to most stable (5). **a** TSSs with least stable paused polymerases exhibit greatest levels of H3K4me3 and NURF301 ChIP signal at TSSs. **b** TSSs with least stable paused polymerases exhibit greater NURF activity downstream of the TSS as revealed by averaged profiling of nucleosome shifts between wildtype and *Nurf301* mutant hemocytes. All promoter classes, irrespective of downstream H3K4me3 show changes in nucleosome position upstream of the TSS (5’ indicated) consistent with TF-mediated recruitment. Only promoters with low pause stability show these downstream of the TSS. **c** Analysis of CAGE tags from wildtype (WT) and *Nurf301* mutant (NURF^-/-^) hemocytes at TSSs ranked by pause stability, reveals loss of NURF affects transcriptional output, with elevated levels of transcription from TSSs with low polymerase stability (Quintiles 1-4) and reduced transcription from TSSs with high polymerase stability (Quintiles 5). **d** Averaged nucleosome flanking TSSs of the resultant quintiles were profiled in wildtype (WT) or *Nurf301* mutant hemocytes. **e** Averaged profile of nucleosome position wildtype (blue) relative to *Nurf301* mutant (orange) were plotted simultaneously for TSSs of the paused polymerase quintiles allowing shifts in nucleosome location to be visualized. NURF-dependent shifts are most obvious on TSSs with higher polymerase turnover (less stable pause sites).

**Supplementary Methods**

***galK* recombineering and gap repair for Nurf301 tagging and mutation**

**Transferring BAC plasmid DNA into SW102**

**BAC plasmids**

BAC clones delivering *Nurf301* genomic DNA (*E(bx)*) with some flanking DNA on both 3' and 5' sides in the BAC vectors are shown in S. Fig. 6. To ensure that the necessary flanking regulatory elements were present, both long and short *Nurf301* genomic constructs were tested.


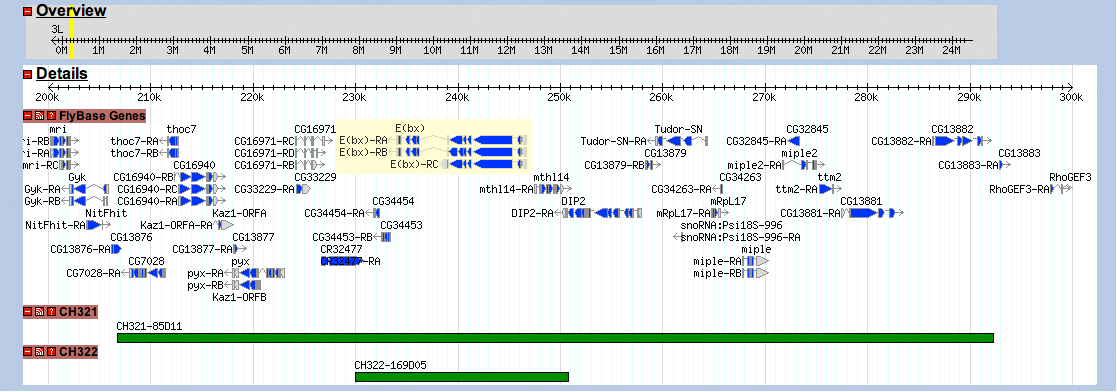


**Supplementary Fig. 6. Map of CH321(85D11) and CH322(169D05) BAC plasmid DNA.** CH321 and CH322 BAC plasmid DNA clones contain *Nurf301* genomic DNA (*E(bx)*) with some flanking DNA on both 3’ and 5’ sides in the BAC vectors as indicated.

**Media compositions**

1x M9 salts was prepared by mixing 6 g Na_2_HPO_4_, 3 g KH_2_PO_4_, 1 g NH_4_Cl and 0.5 g NaCl in 1 L of distilled water as a total volume. For plates 15g/l agar (BD Biosciences) was added prior to autoclaving.

5x M63 salts stock solution was prepared by dissolving 10 g (NH4)2SO4, 68 g KH2PO4 and 2.5 mg FeSO4·7H2O in 1 L distilled water and adjusting to pH 7.0 with KOH.

galK-positive selection plates (M63 medium with 15g/l agar (BD Biosciences), 0.2% + D-galactose (Sigma), 1 mg/ D-biotin (Sigma), 45 mg/l L-leucine (Sigma), 12.5 μg/ml chloramphenicol (Sigma)).

Gal indicator plates (MacConkey agar (BD Biosciences), 1% D-galactose (Sigma), 12.5 μg/ml chloramphenicol (Sigma),

galK counter-selection plates (M63 medium with 15g/l agar (BD Biosciences), 0.2% + glycerol (Sigma), 1 mg/ D-biotin (Sigma), 45 mg/l L-leucine (Sigma), 0.2% 2-Deoxy-D-galactose (DOG, Merck), (12.5 μg/ml chloramphenicol (Sigma)).

**BAC plasmid DNA miniprep**

CH321 and CH322 BAC clones in *E. coli* strain DH10 were streaked onto LB agar plate containing chloramphenicol (25 µg/ml) respectively and incubated for two days at 30ºC. A single colony was picked from each plate and cells were grown in 7.5 ml of LB medium with chloramphenicol (25 µg/ml) in a shaking incubator at 250 rpm for two days at 30ºC. Then the cells were pelleted at 4,500 *g* for five minutes at 4ºC, and the remaining medium was removed. The BAC plasmid DNA was prepared by plasmid miniprep using a QIAprep Spin Miniprep Kit (Qiagen). 50 μl of buffer EB was added to elute DNA at the last step. Air dried CH321 and CH322 BAC DNA pellets were resuspended with 40 µl double distilled water. We stored the DNA samples at 4ºC until needed.

**Electrocompetent cells**

SW102 *E. coli* strain was streaked on to a LB agar plate containing tetracycline (12.5 µg/ml) and incubated overnight at 30ºC. A single colony was inoculated and cultured as a seed culture in 3 ml of LB medium containing tetracycline (12.5 µg/ml) overnight at 30ºC in a shaking incubator (INFORS HT) at 250 rpm. The culture was transferred into 100 ml LB medium without antibiotics and grown for three hours at 30ºC until the OD600 reached 0.6. The cultured cells were cooled on ice slurries for five minutes. The cells were harvested by centrifugation at 3,200 g for 10 minutes at 4ºC and as much as possible of the LB medium was removed. 1 ml of sterilized ice-cold MiliQ water was added to the collected cells to wash, and the cells were resuspended by gentle swirling on ice. We added 1 ml more of sterilized ice-cold MiliQ water before the cells were centrifuged at 3,200 g for 10 minutes at 4ºC. The cells were washed one more time with 1 ml of sterile ice-cold 10% glycerol and pelleted. We removed all of the supernatant and added 125 µl of sterile ice-cold 10% glycerol to resuspend the cells, so that the final volume was equal to 1/800 volume of original cell culture volume.

**Transfer of BAC DNA into the recombinogenic *E. coli* strain SW102**

Isolated BAC DNA was then transferred into the SW102 strain which contains the lambda prophage based recombineering system. SW102 competent cells were prepared as described above, and BAC DNA constructs were delivered into the host strain by performing electroporation as described above. Cells were plated on LB agar plates containing tetracycline (12.5 µg/ml) and chloramphenicol (12.5 µg/ml) as selective markers.

**Supplementary Table 1. Primers for *galK*/TAG containing *Nurf301* 50 bp/500 bp homology arms.**

| **Primer sequence (5’ to 3')** | **Name** | **Description** |
| --- | --- | --- |
| TTGTGCAAAAAATTAAAAATTTTCGCGAAAATGTTTTTGACCAAAGAACACCTGTTGACAATTAATCATCGGCA | *galK*-full-5p | *galK* recombineering DNA templates of *Nurf301*variant A/B |
| CTTTATAAACTATAATCATTTGTTCTAAAGTTTTCAAGGGCTATTATTTCTCAGCACTGTCCTGCTCCTT | *galK*-full-3p |  |
| TTGTGCAAAAAATTAAAAATTTTCGCGAAAATGTTTTTGACCAAAGAACAGAGCAGAAGCTTATCTCCGAG | GSTAP-full-5p |  |
| CTTTATAAACTATAATCATTTGTTCTAAAGTTTTCAAGGGCTATTATTTCCTATTCAGTGACATGAAAGT | GSTAP-full-3p |  |
| TTGTGCAAAAAATTAAAAATTTTCGCGAAAATGTTTTTGACCAAAGAACAGAAATCGGAACTGGTTTTCCG | Halo-full-5p |  |
| CTTTATAAACTATAATCATTTGTTCTAAAGTTTTCAAGGGCTATTATTTCTTCAACCGGAAATCTCTAGAGT | Halo-full-3p |  |
| TAGCCCTTGAAAACTTTAGAACAA | Longarm 3-3 |  |
| AGTCCAATGAGCAGTAAACAAACC | Longarm 3-5 |  |
| GTTCACCAATCGGCTGCGCATCAGCAAGTGAGTAGTTTTCCTGTTGACAATTAATCATCGGCA | *galK*-NurfC-5p | *galK* recombineering DNA templates of *Nurf301*variant C |
| CTGTTCCTATTCCTCATTTATACTTTTACATTATAATTATAATCAGCACTGTCCTGCTCCTT | *galK*-NurfC-3p |  |
| GTTCACCAATCGGCTGCGCATCAGCAAGTGAGTAGTTTTGAGCAGAAGCTTATCTCCGAG | GSTAP-NurfC-5p |  |
| CTGTTCCTATTCCTCATTTATACTTTTACATTATAATTATAACTATTCAGTGACAGTGAAAGT | GSTAP-NurfC-3p |  |
| GTTCACCAATCGGCTGCGCATCAGCAAGTGAGTAGTTTTgaaatcggaactggttttccg | Halo-NurfC-5p |  |
| CTGTTCCTATTCCTCATTTATACTTTTACATTATAATTATAATCAACCGGAAATCTCTAGAGT | Halo-NurfC-3p |  |
| ATAATTATAATGTAAAAGTATAAATGAGG | CLongarm 3-3 |  |
| GCTTAATGTGCTTCCTACTCACT | CLongarm 3-5 |  |

All primers were supplied from Eurofins MWG Operon.


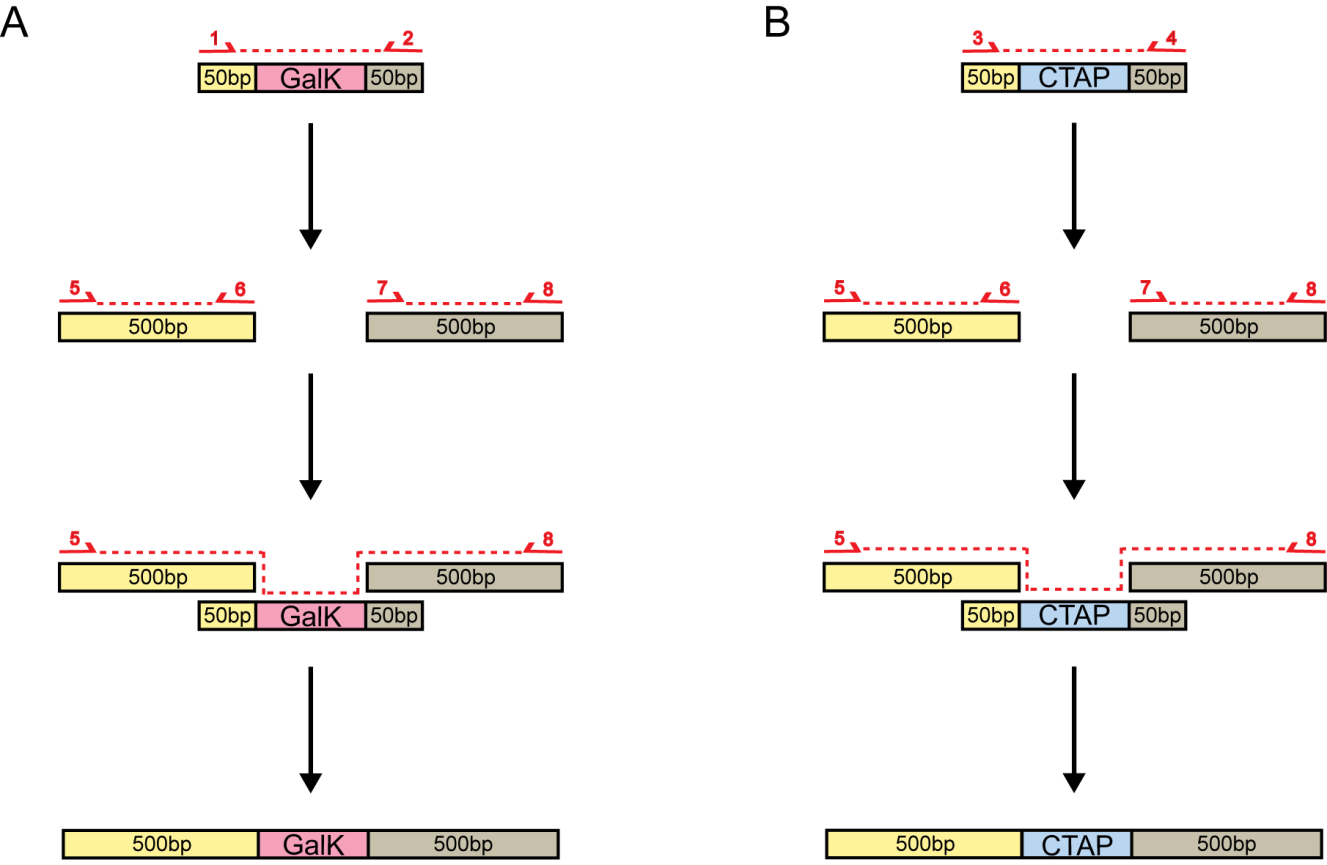


TAG*f3rf3inally CTAPP-ent primer sets shown in S. is required to regulatepe occurs renttion, NURF recruitment and sliding not in*­­­

TAG*f3rf3inally CTAPP-ent primer sets shown in S. is required to regulatepe occurs renttion, NURF recruitment and sliding not in*

TAG*f3rf3inally CTAPP-ent primer sets shown in S. is required to regulatepe occurs renttion, NURF recruitment and sliding not in*

**Supplementary Figure 7. Generation of DNA templates for *galK* recombineering.** (A) The *galK* +/- selection templates and (B) C-terminally tagging of NURF301 templates used 50 bp or 500 bp homology arms on both 3' and 5', so they can be incorporated into the C-terminal of *Nurf301* genomic region during recombination. The primers utilized are listed and numbered as in S. Table 1 above (both NURF full A/B and NURF C isoforms).

**Preparation of the recombination DNA templates**

***galK* positive and negative selection templates**

To generate a galactokinase (*galK*) recombination template DNA with the *Nurf301* full A/B isoform that contains 50 bp length homology arms on both 3' and 5', we conducted PCR (with the *galK*-full-5p and *galK*-full-3p primers described in S. Table 1 and p*galK* plasmid (Addgene) as a template. After the reaction, the 1.2 kb of *galK* PCR product delivering 5'/3' 50 bp homology arms was run on a 1.0% agarose gel electrophoresis and gel purified. 5' and 3' 500 bp homology arm DNA fragments were also prepared by PCR amplification with Longarm 5-5/Longarm 5-3 for 5' end 500 bp homology arm and Longarm 3-5/Longarm 3-3 for 3' end 500 bp homology arm using CH321 BAC plasmid DNA as a template. Subsequently, we performed another PCR to generate a *galK* recombination template DNA with *Nurf301* full A/B isoform 500 bp homology arms on both the 3’ and 5’ ends. Three different templates were required namely: 1) 5’ end 500 bp arm DNA fragment, 2) 3’ end 500 bp arm DNA fragment and 3) the *galK* recombination DNA delivering 50 bp homology arms generated above. The three templates were mixed in equimolar ratios. The final *galK* recombination template DNA with *Nurf301* full A/B isoform 500 bp length homology arms on both the 3’ and 5’ ends was generated by PCR with the Longarm 5-3/3-5 primers as shown in S. Fig. 7. The resultant 2 kb of PCR product was purified by DNA purification using QIAquick Gel Extraction Kit (Qiagen). We performed the same procedures for generation of *Nurf301 C* isoform *galK* recombination templates delivering 50 bp or 500 bp homology arms on both 3' and 5' ends.

**Templates for C-terminally tagged NURF301**

To generate templates for C-terminally tagged recombination of *Nurf301* full A/B isoform with 50 bp length homology arms on both 3’ and 5’ ends, we conducted PCR with the either the GFP-full-5p and GFP-full-3p primers, GSTAP-full-5p and GSTAP-full-3p, or Halo-full-5p and Halo-full-3p, primers described in S. Table 1. For fluorescently tagged proteins the plasmids pEGFP-N1 or pEYFP-N1 (Clontech) were used as templates. For GSTAP-tagged constructs the pCeMM CTAP plasmid (Research Centre for Molecular Medicine of the Austrian Academy of Sciences) was used as a template. For Halo-tagged constructs plasmid encoding the HaloTag (Promega) was used. PCR products delivering 5'/3' 50 bp arms were run on a 1.0% agarose gel and gel purified. Next, we performed another PCR to make final recombination templates with 500 bp length homology arms on both 3’ and 5’ ends. The same 5’ and 3’ 500 bp arm DNA fragments prepared above were used to this reaction. Three different templates were required namely: 1) 5’ 500 bp arm DNA fragment, 2) 3’ 500 bp arm DNA fragment and 3)' the Tag recombination DNA delivering 50 bp homology arms generated above. The three templates were then mixed in equimolar ratios, and a PCR reaction was carried out with the Longarm 5-3/3-5 primers as shown in S. Fig. 6. The final PCR product, approximate size 1.5 kb, was gel purified. We performed the same procedures for generation of *Nurf301* *C* isoform Tag recombination templates delivering 50 bp or 500 bp homology arms on both 3' and 5' ends using the equivalent primer sets shown in S. Table 1.

**Induction of recombineering function in SW102**

SW102 single colonies delivering the CH321/CH322 BAC construct were selected from the plates respectively and cultured in 3 ml LB containing chloramphenicol (12.5 µg/ml) for two days at 30°C. The culture was transferred into 100 ml fresh LB without antibiotics and grown for three hours at 250 rpm at 30°C in a shaking incubator until the OD_600_ reached 0.6 - 0.7. 50 ml of culture was transferred into a sterile falcon tube and reserved at 32°C as an uninduced control. The rest of the culture (50 ml) was incubated for 15 minutes exactly at 42°C in a shaking incubator to induce the expression of the recombineering function. Subsequently, we prepared uninduced and induced SW102 electrocompetent cells as described above.

**Introducing the *galK* containing cassette**

Uninduced and induced competent cells were transformed with 300 ng of *galK* template DNA (*Nurf301* full A/B and C isoforms) with either 50 bp or 500 bp arms by electroporation. After recovery in SOC for one hour at 37°C, 1 ml of cells was transferred into a 1.5 ml Eppendorf tube and centrifuged at 21,000 *g* for 15 seconds at room temperature. All the medium was removed, and cells were resuspended with 1 ml M9 salts. The sample was then pelleted and the supernatant was discarded followed by another two washes with 1 ml M9 salts. This M9 salt wash was necessary to remove any rich medium from the bacterial culture. Finally, 100 μl of the uninduced control and 100 μl of the induced sample with serial dilutions (1/1, 1/10 and 1/100) were plated on *galK*+ selection M63 minimal medium. The plates were incubated for 2-4 days at 30°C. Subsequently, the eight colonies of *galK*+ from each plate were selected and streaked on McConkey agar plate, containing galactose as the sole source of carbon, in order to select against *galK*- contaminating "hitchhikers".

**Removal of the *galK* containing cassette and tagging**

One of the *galK*+ SW102 red colonies from the McConkey agar plate, containing *galK* tagged CH321/CH322 BAC construct, was selected for another round of recombineering to replace the *galK* cassette with tags at either the C-terminus of full length *Nurf301* or at the C-terminus of the *Nurf301 C* variant. The colony cell was cultured, and uninduced/induced electrocompetent cells were prepared as described above. This time, uninduced and induced competent cells were transformed with 300 ng of tag template DNA (*Nurf301* full A/B and C isoforms) with either 50 bp or 500 bp arms by electroporation. Finally, 100 μl of the uninduced control and 100 μl of the induced sample with serial dilutions (1/1, 1/10 and 1/100) were plated on *galK*- counter selection plates. The plates were incubated for 2-4 days at 30°C. Twelve DOG-resistant colonies were analysed by Spe І restriction enzyme digestion and also by PCR to verify the presence of the tags within the *Nurf301* gene.

**C-terminally tagged BAC DNA amplification**

Each of the tagged CH321 full length NURF301, CH322 full length NURF301, CH321 NURF301-C variant and CH322 NURF301-C variant DNA in host SW102 was purified by plasmid DNA miniprep as described above. The DNA constructs were then transformed into the amplification host *E. coli* strain EPI300 (Epicentre) by electroporation. Cells were plated on LB agar plates medium containing chloramphenicol (12.5 µg/ml) as a selective marker. Single colonies were selected from each plate, and seed cell cultures were performed in 3 ml of LB medium containing chloramphenicol (12.5 µg/ml) overnight at 30 ºC in a shaking incubator. Cells were then transferred to 200 ml of LB medium containing chloramphenicol (12.5 µg/ml) and incubated at 30°C until the cell culture reached an OD_600_ of 0.4~0.6. The DNA was amplified by addition of copycontrol induction solution (Epicentre) to a 1x final concentration. The cells were incubated for two hours at 30°C in a shaking incubator. Finally, all tagged NURF301 BAC DNA constructs were purified by Nucleobond Xtra midi Endotoxin Free kit (Macherey-Nagel). At the final step, DNA was dissolved in 100 µl double distilled water by gentle pipetting. The purified DNA samples were visualized by 0.7% agarose gel electrophoresis and quantified using a UV spectrometer (Ultrospec 2100 pro, Amersham).


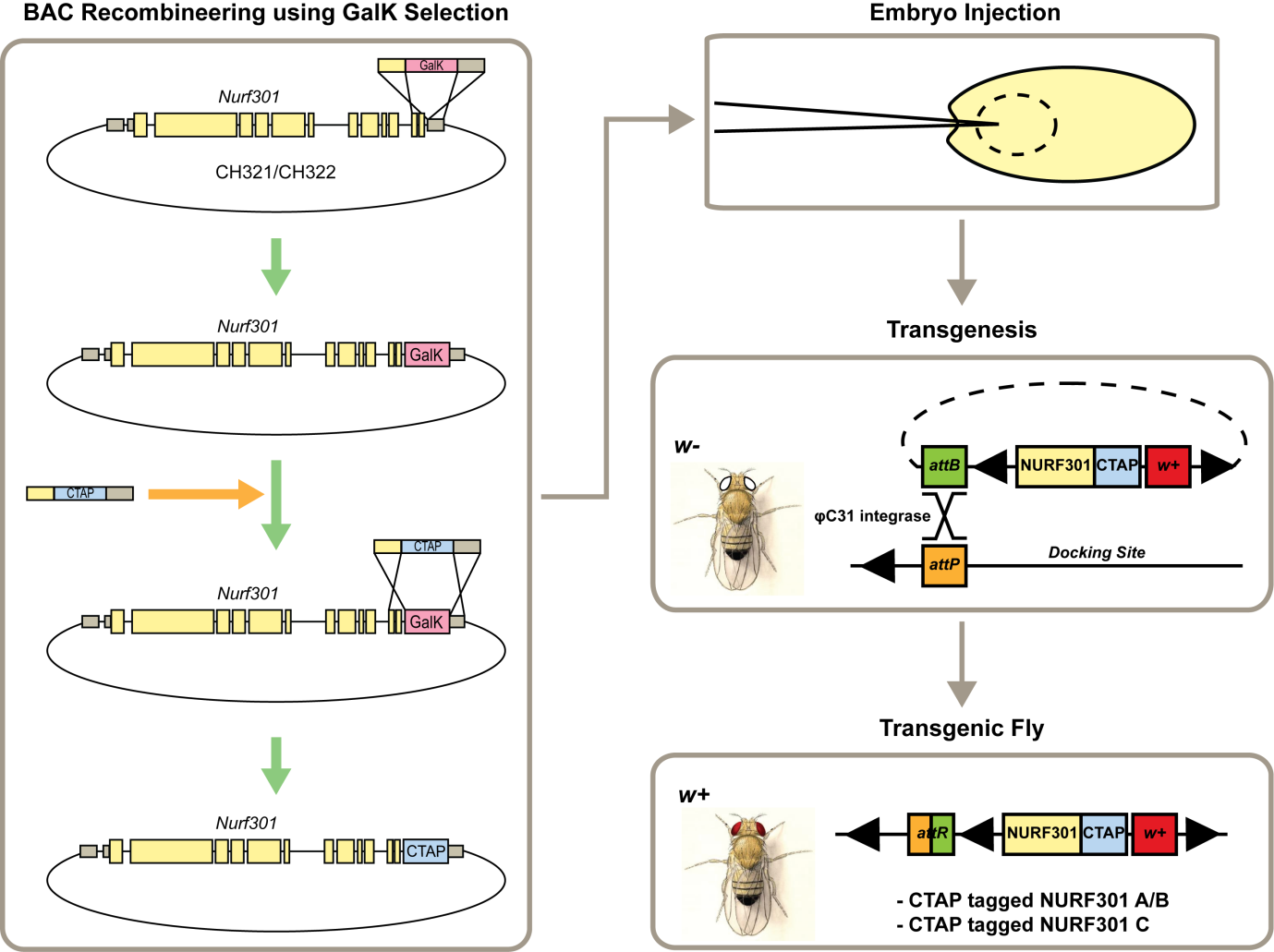


**Supplementary Figure 8.** **Overview of the generation of C-terminally tagged *Nurf301* constructs by using BAC recombineering system and transgenic fly expressing tagged NURF301.** CH321 and CH322 BAC plasmids delivering *Nurf301* genomic DNA (*E(bx)*) were used as templates for recombination, and *y^1^ M{vas-int.Dm}ZH-2A w^*^; M{3xP3-RFP.attP}ZH-86Fb* *Drosophila* strain was prepared for embryo injection.

**Injection of C-terminally tagged NURF301 BAC DNA constructs**

To generate C-terminally-tagged NURF301 transgenic lines, the φC31 integrase system was utilized^2, 3^. DNA was injected into embryos of the strain *y^1^ M{vas-int.Dm}ZH-2A w^*^; M{3xP3-RFP.attP}ZH-86Fb*, which carries an *attP* docking site, at 86F on chromosome 3R and a construct that expresses φC31 integrase in the germline cells. To prepare embryos for injection, approximately 500 young flies (2-3 days) were transferred to an egg-laying cage and fed with yeast paste on apple agar collection plates overnight. The next day, embryos were collected every 20-30 minutes, hand dechorionated and lined up on a glass slide coated with double-sided tape (3M). Embryos were injected with 0.8 µg/µl purified endonuclease free BAC plasmid DNA using an Eppendorf FemtoJet Microinjector and Leitz Labovert FS Inverted Microscope with injection stand and needle holder. Injected embryos were incubated at 18°C for recovery. Larvae were collected after 2-3 days and transferred to fresh vials with food at 25°C. Viable adults were singly mated to *w^1118^* flies of the corresponding sex, and then the progenies were examined for red-eyed transgenic founders (S. Fig. 8).

**Supplementary references**

1. Shao W, Zeitlinger J. Paused RNA polymerase II inhibits new transcriptional initiation. *Nature genetics* **49**, 1045-1051 (2017).

2. Bischof J, Maeda RK, Hediger M, Karch F, Basler K. An optimized transgenesis system for Drosophila using germ-line-specific phiC31 integrases. *Proc Natl Acad Sci U S A* **104**, 3312-3317 (2007).

3. Venken KJ, He Y, Hoskins RA, Bellen HJ. P[acman]: a BAC transgenic platform for targeted insertion of large DNA fragments in D. melanogaster. *Science (New York, NY)* **314**, 1747-1751 (2006).
