# Supplementary material for: Combinatorial histone modifications direct ATP-dependent chromatin remodeling by NURF to promoter-proximal nucleosomes
Source: Nucleic Acids Res. 2026 May 21;54(10):gkag494. doi: 10.1093/nar/gkag494 (PMC13191287; doi:10.1093/nar/gkag494)
Supplement: gkag494_Supplemental_Files [file gkag494_supplemental_files.zip › Supplementary_Movie-Legends.docx]

**SUPPLEMENTARY MOVIE LEGENDS**

**Movie 1:** Single particle tracking of individual nucleus showing Halo-tagged full-length NURF301-A labelled using 0.5nM JF646 HALO-ligand and imaged at 500ms intervals to motion blur unbound particles and resolve stably-bound NURF complexes..

**SUPPLEMENTARY MOVIE LEGENDS**

**Movie 2:** Single particle tracking of individual nucleus showing Halo-tagged C-terminally trun cated NURF301-C labelled using 0.5nM JF646 HALO-ligand and imaged at 500ms intervals to motion blur unbound particles and resolve stably-bound NURF complexes..
